# The buzz in the field: the interaction between viruses, mosquitoes, and metabolism

**DOI:** 10.3389/fcimb.2023.1128577

**Published:** 2023-04-26

**Authors:** Oshani C. Ratnayake, Nunya Chotiwan, Karla Saavedra-Rodriguez, Rushika Perera

**Affiliations:** ^1^Center for Vector-borne Infectious Diseases, Dept. of Microbiology, Immunology and Pathology, Colorado State University, Fort Collins, CO, United States; ^2^Chakri Naruebodindra Medical Institute, Faculty of Medicine Ramathibodi Hospital, Mahidol University, Samut Prakan, Thailand

**Keywords:** *Aedes aegypti*, mosquito, Wolbachia, metabolism, lipids, dengue, Zika, virus

## Abstract

Among many medically important pathogens, arboviruses like dengue, Zika and chikungunya cause severe health and economic burdens especially in developing countries. These viruses are primarily vectored by mosquitoes. Having surmounted geographical barriers and threat of control strategies, these vectors continue to conquer many areas of the globe exposing more than half of the world’s population to these viruses. Unfortunately, no medical interventions have been capable so far to produce successful vaccines or antivirals against many of these viruses. Thus, vector control remains the fundamental strategy to prevent disease transmission. The long-established understanding regarding the replication of these viruses is that they reshape both human and mosquito host cellular membranes upon infection for their replicative benefit. This leads to or is a result of significant alterations in lipid metabolism. Metabolism involves complex chemical reactions in the body that are essential for general physiological functions and survival of an organism. Finely tuned metabolic homeostases are maintained in healthy organisms. However, a simple stimulus like a viral infection can alter this homeostatic landscape driving considerable phenotypic change. Better comprehension of these mechanisms can serve as innovative control strategies against these vectors and viruses. Here, we review the metabolic basis of fundamental mosquito biology and virus-vector interactions. The cited work provides compelling evidence that targeting metabolism can be a paradigm shift and provide potent tools for vector control as well as tools to answer many unresolved questions and gaps in the field of arbovirology.

## 1 Introduction

### 1.1 Arboviruses are a significant disease burden to human populations

Arboviral infections are becoming increasingly aggressive on a global scale due to climate change, global travel and the development of insecticide resistance in vectors. They are vectored by mosquitoes, ticks, sandflies or biting midges. Among these, mosquito-borne viruses contribute heavily to the disease burden in human populations especially in developing countries (Franklinos et al., 2019). Flaviviruses such as dengue, Zika and West Nile and alphaviruses such as chikungunya are amongst the most common mosquito-borne viruses causing human disease. Dengue viruses (DENVs) are responsible for ~400 million infections each year and more than a quarter of the global population lives in endemic areas (Bhatt et al., 2013). Zika virus (ZIKV) has caused severe epidemics and according to the World Health Organization (WHO), a total of 86 countries and territories have reported ZIKV cases to date (World Health Organization, 2022). In addition to being transmitted by the mosquito vector, ZIKV can be transmitted from mother to fetus during pregnancy, through sexual contact, blood transfusions and organ transplants thus widening its transmission capacity. It is estimated that during the last epidemic in 2015-2016, approximately 1.5 million people were infected by ZIKV in Brazil with over 3,500 microcephaly cases reported (European Center for Disease Prevention and Control, 2015). West Nile virus (WNV) is another important agent causing disease in both humans and horses and is the most common etiological agent of viral encephalitis (Chancey et al., 2015). There is currently no vaccine available for WNV. Besides these flaviviruses, chikungunya virus (CHIKV) is an alphavirus that has caused severe outbreaks in Asia, Africa, Americas, and Europe making it a public health concern globally (World Health Organization, 2020). As of October 2022, nearly 3,400,000 chikungunya cases and 70 deaths have been reported globally with Brazil having the most cases (European Center for Disease Prevention and Control, 2022). During the massive outbreak in 2005-2006 in La Reunion Island, the virus acquired the ability to transmit *via* its secondary vector, *Aedes albopictus* due to an amino acid change in the E1 glycoproteinintheEast-

Central-South African genotype of the virus. In addition, other mutations in the E1 and E2 glycoproteins have further increased mosquito infectivity of the virus (Tsetsarkin et al., 2007).

### 1.2 Mosquitoes are the primary vector of medically relevant arboviruses

*Ae. aegypti* (Diptera: Culicidae) is the major mosquito vector that transmits the viruses discussed above. These mosquitoes inhabit various regions of the world including both tropical and subtropical areas across several continents including Asia, Africa, North and South America, Europe and Australia (Kraemer et al., 2015). Since they are anthropophilic, these mosquitoes are well adapted to rapid urbanization and prefer artificial water containers for egg laying (Scott and Takken, 2012; Kraemer et al., 2015). Interestingly, the geographical distribution of the mosquito vector is temperature dependent (Brady et al., 2012; Brady et al., 2014; Kraemer et al., 2015) and it has been predicted that the mosquito habitats will be expanded to currently more temperate regions due to climate change (Khormi and Kumar, 2014; Mweya et al., 2016). Therefore, it is anticipated that even larger human populations will be exposed to these disease carrying vectors in the future.

### 1.3 Metabolism is at the forefront of mosquito development and biology

Living organisms are vastly diverse. Every organism has signature characteristics in morphology, anatomy and physiology. Further, there is significant diversity in factors such as behavior and ecology. On the contrary, organisms are also remarkably analogous to each other based on fundamental traits at the molecular level. These similarities are basically mirrored in metabolism and biochemical mechanisms of inheritance (Robert Burger et al., 2021). Metabolites are universal molecules that do not vary across species or ecological barriers. These molecules are reflective of the output of genetic expression (DNA/RNA/protein interactions). Metabolic adaptations occur within an organism when in need of maintaining energy homeostasis and development under different environmental stimuli (Koyama et al., 2020). Therefore, metabolism is intimately associated with the biology of an organism. Investigating mosquito metabolism can provide the blueprint of a mosquito’s response to stimuli such as a viral infection, the changing microbiome, insecticide resistance and environmental changes. Importantly, metabolites can be traced back to identify the genotype of a particular phenotype helping to understand the molecular basis of biochemical responses.

Lipid metabolism in mosquitoes has been studied since the mid-1900s. Due to the lack of advanced technologies, these studies focused on the response of mosquito lipids to different diets, the conversion of food to fat, storage of fat in the fat body of the insects, the utilization of fat for energy during flight, metamorphosis, starvation, and the deposition of fat for oogenesis. The recent availability of the genome sequences of certain mosquitoes, advanced molecular biology techniques and the advent of systems biology approaches especially techniques in metabolomics have helped us understand lipid metabolism at a molecular level in mosquitoes as well as other insects. The first half of this review will focus on the findings from the late 1900s to the early 2000s, on the utilization of lipids in several physiological processes of mosquitoes. The second half of this review will focus on the discovery (or re-discovery) of mosquito lipids from the molecular biology/omics era.

## 2 The mosquito life cycle is intimately associated with metabolic processes

**Figure 1:**
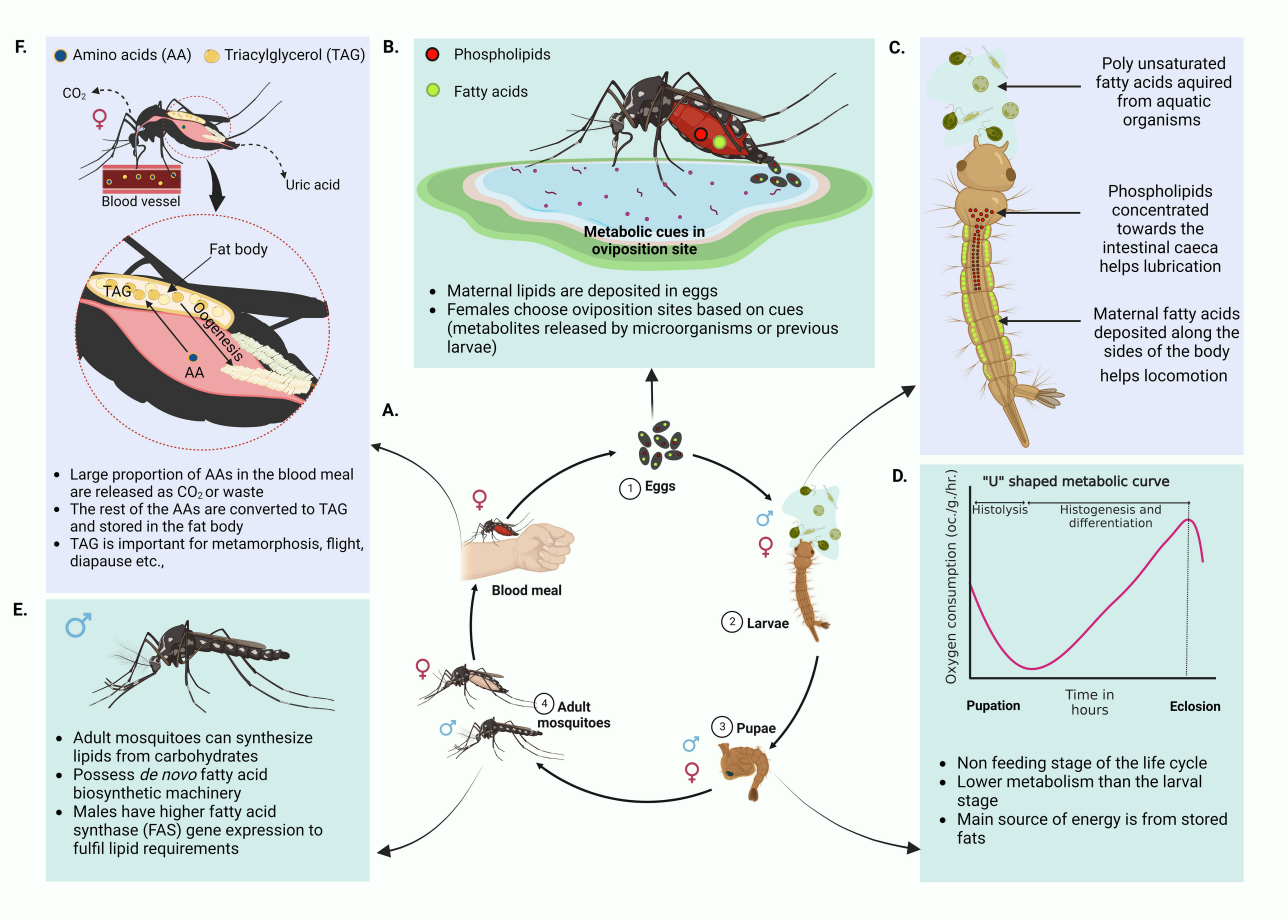
Mosquito life cycle and related metabolism. **(A)** Life cycle of the mosquito. Four major stages of the mosquito life cycle, eggs, larvae, pupae and adults. Male mosquitoes feed on nectar and other sugar sources whereas the female mosquitoes require a vertebrate blood meal for egg maturation. **(B)** Deposition of maternal lipids in eggs and selection of oviposition sites. Majority of lipids in mosquito eggs come from maternal lipid deposits (Ziegler, 1997; Canavoso et al., 2001; Ziegler and Ibrahim, 2001; Atella and Shahabuddin, 2002; Sushchik et al., 2013). These lipids are generated post blood meal. Only a small proportion of egg lipids represent lipids generated from a sugar meal. Maternal lipid deposits aid the survival of the neonate larvae. Female mosquitoes exploit a range of environmental cues, especially metabolites in the habitats to select good oviposition sites (Perry and Fay, 1967; Ikeshoji et al., 1975; Ikeshoji et al., 1979; Bentley and Day, 1989; Davis, 1976) **(C)** Differential distribution of maternal lipids in neonate larvae. Maternal lipid distribution commences at the embryonic stages. Maternal phospholipids concentrated towards the intestinal caeca of the larvae assists lubrication and assimilation of nutrients from the diet. Maternal fatty acids are deposited along the sides of the body, especially in association with muscles. These help the neonate larvae locomote in search of food (Atella and Shahabuddin, 2002; Sushchik et al., 2013). **(D)** “U” shaped metabolic curve of pupal stage. During this non feeding life stage, pupae use energy mainly from stored fats. If metabolic rate is measured with respect to rate of oxygen consumption, pupae show the least metabolic rate and have a characteristic “U” shaped curve (Chapman, 2013). **(E)** Adult mosquito biology. Both male and female mosquitoes can synthesize lipids from sugar meals. Females can do this faster than males. They also have *de novo* fatty acid biosynthetic machinery. Males have more active fatty acid synthase gene expression. This is reflective of dependence on *de novo* fatty acid synthesis to fulfill lipid requirements that are not fulfilled by the high sugar diet (Jenkin et al., 1975; Sushchik et al., 2013; Chotiwan et al., 2022) **(F)** Utilization of amino acids in the blood meal by female mosquitoes. A major proportion of the blood meal amino acids are oxidized to CO_2_ or excreted as uric acid while the remaining portion is converted to triglycerides (TAGs) and stored in the mosquito fat body (Lehane, 1991; Zhou et al., 2004a; Horvath et al., 2021). Deposited TAGs are used in oogenesis, metamorphosis, prolonged flight and other physiological processes. *(Created with BioRender.com)*.

The mosquito life cycle has four major stages (**Figure 1A**, 1-4): eggs, larvae, pupae and adults. Each stage has a different morphology, habitat, behavior, food source, and thus is exposed to different metabolic sources driving differential usage of metabolites. Additionally, each stage of the mosquito life cycle is regulated to provide optimum resources for the next developmental phase. Therefore, the conditions faced by the immature phases of the mosquito drive reproductive success, longevity, and vector competence of the adult mosquito. Communication between different metabolic pathways that are active at each life stage may be key to this nutritional continuity.

Energy and nutritional requirements of each life stage depend on the acquired meal which provides a unique repertoire of nutrients. As reviewed by Rivera-Pérez, Clifton and Noriega, 2017 nutritional requirements of a mosquito can be classified into two groups, macronutrients (Carbohydrates, fatty acids and amino acids) and micronutrients (vitamins, salts, sterols and metals) (Rivera-Pérez et al., 2017). In general, adult mosquitoes demand more energy for active flight and reproduction (Nayar and Van Handel, 1971). Lipids serve as the ideal energy source due to their high caloric value per amount of substrate as well as the ease of storage as anhydrous triglycerides (Downer and Matthews, 1976). Further, lipid metabolism plays a vital role in mosquito vitellogenesis and egg generation. Female mosquitoes transfer a major portion of lipids acquired *via* a sugar meal (prior to a blood meal) into the ovaries (Briegel et al., 2002). Due to these reasons, lipid metabolism plays a key role in efficient nutrient utilization in mosquitoes.

### 2.1 Eggs

Eggs are laid in water (oviposition). Being anautogenous insects (ex: *Ae. aegypti*), female mosquitoes require a vertebrate blood meal in order to produce eggs (Clements, 1992). Studies on eggs of other insects like *Manduca sexta* (tobacco hornworm) have shown that approximately 40% the dry mass of eggs represents lipids. Most of these lipids are acquired through maternal depositions and only 1% is generated in the egg (Canavoso et al., 2001). Provided that lipids have various functions, distribution of lipids in suitable tissues in the developing embryo is important for the emerging neonates (Atella and Shahabuddin, 2002).

After consumption of an adequate blood meal to facilitate ovarian development, female mosquitoes engage in the quest of finding suitable oviposition sites. Different mosquito species have varying preferences for oviposition sites. *Ae. aegypti* mosquitoes prefer freshwater habitats for egg laying while some species of *Culex* lay eggs in a wide range of sites from salt marshes to artificial containers. As reviewed by Bentley and Day, mosquitoes select their oviposition sites based on chemical and physiological cues at the site (**Figure 1B**) (Bentley and Day, 1989). Experiments on gravid colony *Ae. aegypti* mosquitoes have reported olfactory responses to fatty acid esters (Perry and Fay, 1967). A different study (Davis, 1976) reported one of the fatty acid esters, methyl propionate, as an active chemoattractant in oviposition. Additional studies have reported the influence of metabolites such as 7,11-dimethyloctadecane produced by the bacterium *Pseudomonas aeruginosa* as an oviposition attractant (Ikeshoji et al., 1975; Ikeshoji et al., 1979). According to a review by Bentley et al., there are multiple cues that are either of larval, pupal or adult origin that influence oviposition site selection in mosquitoes. These could be metabolites released from the previous life stages signaling the newly gravid mosquitoes the suitability of the site for safe oviposition (Bentley and Day, 1989).

### 2.2 Larvae

Eggs hatch to produce larvae that undergo four instar stages before developing into a non-feeding pupa. Neonate larvae acquire most of the lipids from the mother through the maternal deposition of lipids in eggs (Ziegler, 1997; Ziegler and Ibrahim, 2001; Atella and Shahabuddin, 2002). As discussed previously, there is a considerable functional variability between lipids. Sequestering appropriate lipids in suitable sites in the embryo is critical for the health of neonates (Atella and Shahabuddin, 2002). Using fluorescently labeled fatty acids and phospholipids, Atella and Shahabuddin were able to track the distribution of maternal lipids in developing mosquito eggs and larvae. They found that fatty acids were distributed along the sides of the larval body especially where the muscles are located, while phospholipids aggregated along the intestinal gastric caeca (**Figure 1C**) (Atella and Shahabuddin, 2002). The authors justify this distribution mentioning the different functions owned by the lipids. Fatty acids deposited alongside the larvae body, especially in association with muscles are assumed to provide energy to support locomotion and rapid movements of newly emerged larvae to find food. Maternal phospholipids that are accumulated in the motile gastric caeca secrete lubricants into the lumen of the gut. These are possibly aiding the neonate larvae in assimilating ingested food. Larvae further acquire lipids, especially the essential polyunsaturated fatty acids, from aquatic food sources such as diatoms and algae (Sushchik et al., 2013). These lipids are required for the proper functioning of innate immunity, developmental processes, and the ability to fly in their adult stage (Dadd and Kleinjan, 1979; Dadd et al., 1987; Stanley and Miller, 2006). Fatty acids that are acquired during larval stages are transferred to the adult stages. However, during metamorphosis, fatty acid conversions occur where eicasopentanoic acid (EPA) and Arachidonic acid (AA) are transferred to the adult mosquito from the triacylglycerol (TAG) stores of the larvae to generate more polar lipids (Sushchik et al., 2013). Further, studies have shown how larval diet can alter vector competence in the adult *Ae. aegypti* mosquito (Nasci and Mitchell, 1994; Muturi et al., 2011). Female *Ae. aegypti* mosquitoes developing from larvae that are fed with a high nutrient diet have presented larger body size which is related with a greater metabolic reserve (Briegel, 1990). The feeding success of these female mosquitoes on vertebrate hosts is also significantly greater. This suggests that better nutrition at larval stages can impact the adult vectorial capacity. Moreover, studies by Silva et al., 2021 has shown how higher larval rearing densities can elevate stored TAG levels within adult mosquitoes and also influence the size and fecundity of the mosquito (Silva et al., 2021).

### 2.3 Pupae

The pupae stage is a non-feeding period solely relying on energy stored at the larval stage. These energy reserves thus determine the ability of a newly emerged adult to survive, reproduce and transmit disease. If metabolic rate is determined by the rate of oxygen consumption of the organism, pupae show the least rate in comparison to larvae and the adult stages (Chapman, 2013). However, this metabolic rate fluctuates with time where it is initially high and then drops before it rises again by the time of eclosion (Chapman, 2013). The trend of metabolic variation is known to have a characteristic ‘U’ curve indicating the fall of metabolic rate initially at histolysis followed by an increase at histogenesis and differentiation (**Figure 1D**). Interestingly, the main source of energy is gained *via* fats (supplemented with a smaller portion of carbohydrates) during the pupal period (Chapman, 2013).

### 2.4 Adult mosquitoes

Adult mosquitoes are able to synthesize lipids from carbohydrate (sugar) meals (Ziegler and Ibrahim, 2001). Both males and females possess *de novo* fatty acid biosynthesis machinery, such as fatty acid synthase (FAS) and Δ-9 fatty acid desaturase enzymes, but males have higher FAS gene expression than females to fulfil lipid requirements (**Figure 1E**) (Jenkin et al., 1975; Sushchik et al., 2013; Chotiwan et al., 2022). By feeding on sugar meals alone, the females are capable of increasing their lipid content up to 300µg within 5 days (Ziegler and Ibrahim, 2001).

**Figure 2:**
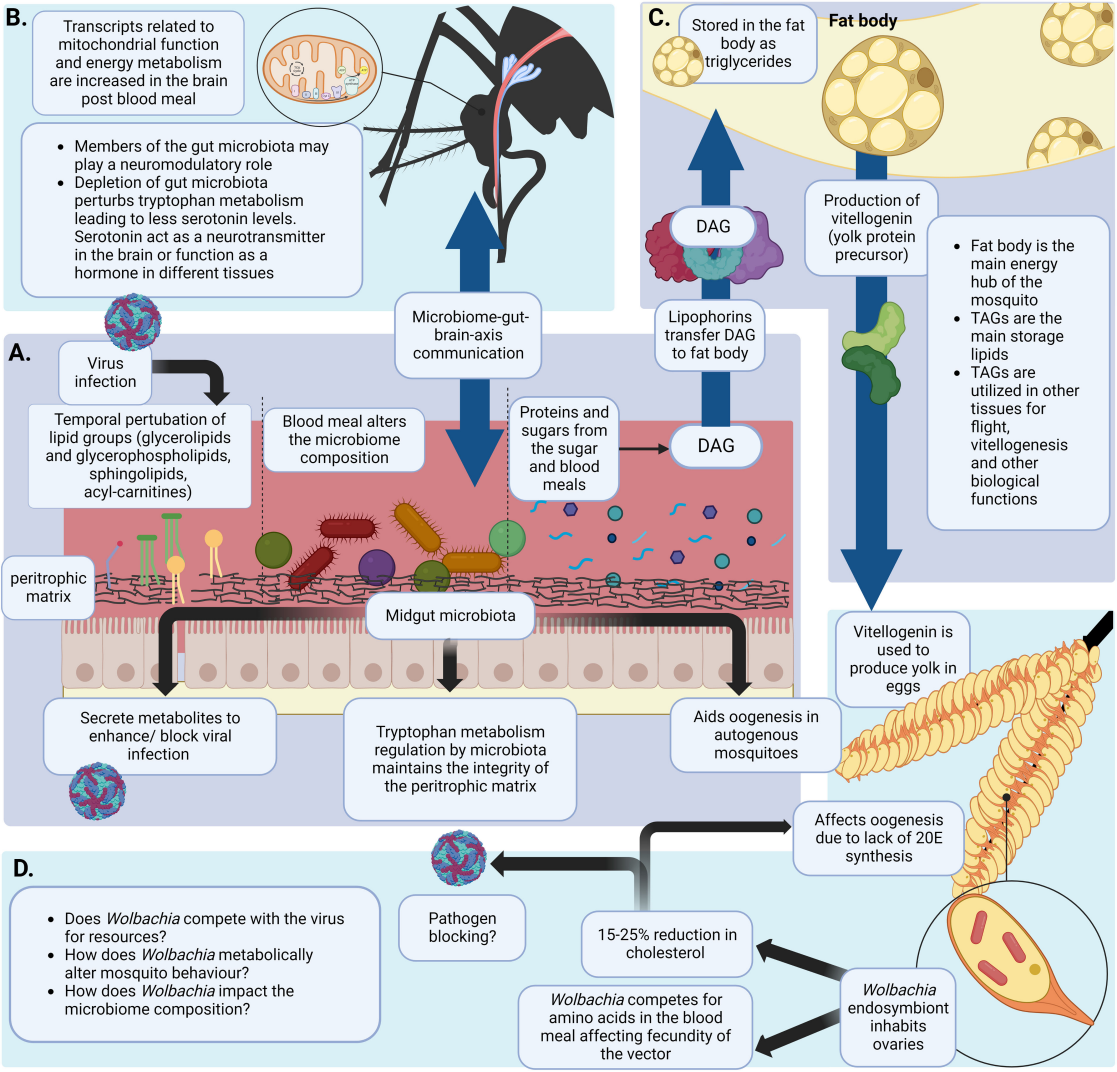
Blood meal induced metabolic changes in the mosquito. **(A)** The mosquito midgut acts as a major hub in metabolism related processes. It has a unique structure and a metabolic landscape that can be altered due to the blood meal. A virus entering the midgut *via* a blood meal and establishing infection can temporally alter the lipid landscape of the midgut (Chotiwan et al., 2018). The blood meal can also alter the gut microbiota diversity and composition. Gut microbiota are involved in metabolic processes like maintaining the integrity of the peritrophic matrix by regulating tryptophan metabolism. This is important to prevent *Plasmodium* spp. Infections (Okech et al., 2006; Lima et al., 2012; Feng et al., 2021; Bottino-Rojas et al., 2022). Microbiota also plays a role in enhancing/blocking viral infections by secreting metabolites or altering metabolic homeostasis (Apte-Deshpande et al., 2012; Ramirez et al., 2012; Apte-Deshpande et al., 2014; Ramirez et al., 2014). Nutritional resources required for egg production by autogenous mosquitoes are fulfilled by gut microbiota of the mosquito (Coon et al., 2016) **(B)** Intake of a protein rich blood meal activates the ‘metabolic switch’ in female mosquitoes. Following a blood meal brain transcripts related to energy metabolism and mitochondrial function were observed to be increased (Das De et al., 2022). Gut microbiome plays a significant role in gut-brain-axis of communication (Das De et al., 2022) **(C)** Proteins and sugars ingested in a blood and/or a sugar meal are digested and transported to the fat body of the mosquito by lipophorins. Yolk protein precursor-vitellogenin is produced using stored TAGs in the fat body. These proteins are transported to the ovaries for oogenesis (Ziegler and Vanantwerpen, 2006; Arrese and Soulages, 2010; Gabrieli et al., 2014; Wang et al., 2017) **(D)** *Wolbachia* is an endosymbiont mainly inhabiting the ovaries of the mosquito (Hilgenboecker et al., 2008; Zug and Hammerstein, 2012). *Wolbachia* is known to deplete cholesterol in the ovaries which is a precursor for 20E synthesis. This detrimentally affects oogenesis of the mosquito. Moreover, *Wolbachia* is shown to compete with the mosquito vector for amino acids ultimately affecting fecundity of the mosquito (248,25o,252). *(Created with BioRender.com)*.

A blood meal taken by a female mosquito can induce a pronounced metabolic change in its physiological status (**Figure 2A**). This phenomena is known as a ‘ metabolic switch’ (Das De et al., 2022). Das De et al. compared the transcriptome of sugar-fed and blood-fed mosquitos using RNA-seq and showed that feeding blood (a high protein diet), induced expression of transcripts in the brain that are related to mitochondrial function and energy metabolism (**Figure 2B**) (Das De et al., 2022). Blood meals can also serve as an indirect source for lipids. In blood, lipids compose only about 4% of the nutrients (Lehane, 1991). Although no direct evidence has shown that the mosquito midgut epithelium can directly absorb lipids from the blood meal, increases in the expression of the genes that encode the proteins that absorb lipids from food, fatty acid binding protein and long chain fatty acid transport protein, have been reported (Sanders et al., 2003). The other 95% of nutrients in the blood meal are protein (Lehane, 1991). Using [^14^C]-labeled protein meals, Zhou et. al., have shown that approximately 30% of blood meal amino acids were oxidized to CO_2_ or excreted as waste (**Figure 1F**) (Zhou et al., 2004a). To determine how female Ae. aegypti mosquitoes detoxify ammonia that is generated during the oxidation of amino acids in a blood meal, mosquitoes were fed with labeled ^15^NH_4_Cl. The labeled ^15^N was traced in the whole mosquito body using electrospray ionization (ESI)-mass spectrometry and stable label isotope tracing (Horvath et al., 2021). The study showed that ^15^N was rapidly incorporated into glutamine (Gln) *via* glutamine synthase (GS) and with the aid of other enzymes, additional N-containing metabolites were generated in the mosquito (Horvath et al., 2021). However, 16% of the meal was converted to TAG, the storage lipid (Zhou et al., 2004a). The expression of several genes involved in lipid synthesis also increased after the blood meal was taken (Sanders et al., 2003). This finding provides more evidence that the blood meal can serve as a source of lipid reserves in mosquitoes. The reserve lipids are required for several physiological processes, such as oogenesis, metamorphosis, diapause, and prolonged flight (Zhou and Miesfeld, 2009; Arrese and Soulages, 2010; Sushchik et al., 2013). They also serve as a source for fatty acids which are precursors for synthesizing eicosanoids, pheromones, glycerophospholipids (GPs) and wax (Arrese and Soulages, 2010).

### 2.5 Lipid storage and mobilization

In the adult mosquito, the fat body is the central location for lipid synthesis, storage and degradation for energy production (**Figure 2C**) (Arrese and Soulages, 2010). It is an organ composed of loose tissues distributed throughout the insect body, lining the underneath of the cuticle and surrounding the gut and reproductive tissues (Dettloff et al., 2001). The majority of the cells in the fat body are adipocytes. These cells contain numerous lipid droplets which serve as the center of cellular lipid storage and energy metabolism (Olofsson et al., 2009). More than 50% of the dry weight of the fat body are lipids (Ziegler, 1991). The fat body also stores carbohydrates in the form of glycogen which constitutes about 25% of the dry weight. The rest of the carbohydrates (> 50% of intake glucose in *Ae. aegypti*) are oxidized or converted to lipids (Ziegler, 1991; Zhou et al., 2004b). This organ also serves as a source for synthesizing most of the hemolymph proteins. These proteins include lipophorin, the protein that is responsible for transporting lipids between cells or tissues, and vitellogenin, the protein that is required for egg maturation during oogenesis (Ziegler and Vanantwerpen, 2006).

Nutrients that are absorbed in the gut are transported to the fat body and converted to glycogen and lipids (Arrese and Soulages, 2010). Muscle cells only contain a small amount of energy reserves. As a result, the energy required for prolonged flight is provided by the fat body (Kaufmann and Brown, 2008). Less than 1% of lipids in eggs are locally synthesized. More than 80% of lipids in eggs are transferred from the fat body (**Figures 2C, D**) (Ziegler, 1997; Ziegler and Ibrahim, 2001).

Lipophorin is the main hemolymph lipoprotein. It plays a role as a reusable shuttle transporting lipids between tissues (Ziegler and Vanantwerpen, 2006). Similar to human high-density lipoprotein (HDL) and low-density lipoprotein (LDL), there are high- and low-density lipophorins (HDLp and LDLp) in insects. LDLp contains up to 63% of the lipids, while HDLp contains 30-50% of the lipids (Beenakkers et al., 1985). Apolipophorin I and II are integral components of the lipophorin particles whereas Apolipophorin III is transiently associated with the lipophorin particle (Van der Horst and Ryan, 2017). Lipophorins in most insects are enriched in diacylglycerol (DAG). However, lipophorins in mosquitoes and some other dipterans, but not *Drosophila melanogaster* (*D. melanogaster*) are enriched in triacylglycerol (Pennington et al., 1996; Pennington and Wells, 2002). The mechanism/s of lipid uptake from lipophorins into the oocytes are still unclear. Both receptor-mediated endocytosis of the intact lipoprotein particles and extracellular hydrolysis of lipids from the lipoprotein core have been observed (Ziegler and Vanantwerpen, 2006).

### 2.6 The gonadotropic cycle

Female mosquitoes require a considerable amount of energy and intense metabolic support during reproduction. The gonadotrophic cycle (egg production cycle) of an *Ae. aegypti* female is regulated by altering titers of two major hormones, juvenile hormone (JH) and a steroid hormone called 20-hydroxyecdysone (20E) (Attardo et al., 2005; Roy et al., 2015). The gonadotropic cycle has two phases. In the first phase, the posteclosion (PE) phase, JH regulates the development of the mosquito which drives physiological functions related to egg maturation and blood digestion. The female mosquito is physiologically prepared for blood meal digestion and egg maturation by this hormone (Wang et al., 2017; Ling and Raikhel, 2021). The fat body and ovaries need to be exposed to JH in order for the synthesis and accumulation of yolk protein precursor vitellogenin (Vg) (Gwadz and Spielman, 1973) following PE period (previtellogenic maturation), the vitellogenic phase starts with the mosquito taking a blood meal. During this post blood meal phase (PBM), the titers of JH are reduced while 20E titers are increased (Arrese and Soulages, 2010). Cholesterol ingested in a blood meal acts as a precursor of 20E synthesis (Clayton, 1964; Ekoka et al., 2021). Cholesterol stored in the prothoracic glands of the mosquito larvae and pupae can also be used to synthesize 20E (Jenkins et al., 1992). 20E regulates and supports blood meal digestion and egg development in female mosquitoes (Gabrieli et al., 2014; Wang et al., 2017). Interestingly, pathways related to carbohydrate metabolism were shown to be upregulated during the peak of 20E synthesis in females (18-24hPBM) (Hou et al., 2015). Later, Dong et al., have showed that 20E regulated the carbohydrate metabolism through a nuclear transcription factor HR38 (Dong et al., 2018). Additionally, studies by Hou et al. have demonstrated how major carbohydrate metabolic pathways (glycolysis, glycogen and sugar metabolism and the citrate cycle) were considerably downregulated in the mosquito fat body at PE (Hou et al., 2015). However, these pathways were upregulated at the PBM stage. In addition, TAG levels were also decreased at PE but elevated PBM.

Lipids that are synthesized in the fat body are transported and deposited in eggs. These lipids contribute to about 35% of the weight of *Ae. aegypti* oocytes (Troy et al., 1975). It should be noted that lipids that are synthesized from carbohydrate meals are not sufficient to trigger the maturation of oocytes. Blood meals, or to be specific, amino acids in the meal, are needed to trigger the release of vitellogenin stimulating hormone in the ovaries to initiate the maturation process of the oocytes (Hagedorn et al., 1979). Accumulation of lipids in the oocytes starts only after a blood meal is taken (Troy et al., 1975). Although ovaries are capable of synthesizing complex lipids, especially GPs, less than 1% of locally synthesized lipids were found in the egg (Ziegler and Vanantwerpen, 2006). Using radioactively labeled lipids, Ziegler et. al., found that the majority of the lipids in eggs were TAG that was transferred from the fat body (Ziegler, 1997; Ziegler and Ibrahim, 2001; Sanders et al., 2003).

*Ae. aegypti* possess a mechanism to maintain metabolic homeostasis during the gonotrophic cycle. Zhou et al., did not observe differences in lipid and protein content and the number of eggs laid from females that underwent starvation before a blood meal (Zhou et al., 2004b). However, they observed significantly lower lipid and glycogen content in the mother after the eggs were laid. This indicates a trade-off between fecundity of the mother and the quality of the eggs. Although a significant portion of lipids accumulating in the oocytes from the first gonotrophic cycle comes from larval food and pre-existing maternal stores (Zhou et al., 2004b), the ability to *de novo* synthesize fatty acids is still important to produce viable eggs. Transient knockdown (KD) of two key enzymes in the *de novo* fatty acid biosynthesis pathway, acetyl-CoA carboxylase (ACC) and fatty acid synthase (FAS), caused a significantly lower number of eggs in the first gonotrophic cycle (Alabaster et al., 2011). Eggs that were produced from ACC-deficient mosquitoes also lacked eggshells and were nonviable.

## 3 Mosquito immunity and metabolism

Mosquitoes mostly depend on innate immunity. Additionally, innate immune priming in mosquitoes can lead to memory like responses in mosquitoes. Mosquitoes release lipoxin/lipocalin complex as a result of immune priming (Ramirez et al., 2015). Since they are constantly exposed to a variety of microorganisms in varying habitats as well as blood meal sources, the mosquito innate immune system is well adapted to initiate a strong immune response against these foreign entities. Three major immune signaling pathways have been identified in mosquitoes; the Toll, Immune Deficiency (IMD) and Janus/kinase and signal transducers and activators of transcription (JAK-STAT). Besides these immune signaling pathways, the RNA interference (RNAi) pathway also plays an important role during antiviral defense although it is not considered as a classical immune signaling pathway.

Several studies in *Drosophila melanogaster* provide evidence on how metabolism and immunity are related in the fly. Activation of the IMD pathway in the fat body of *D. melanogaster* is associated with modifications in host metabolism. In a transcriptional analysis in *Drosophila*, activation of IMD pathway resulted in changes in expression of metabolism related genes of the fly. For example, genes responsible for the insulin signaling pathway and TOR (Target of Rapamycin) that responds to constant environmental changes and maintains energy, growth and developmental homeostasis of the fly were observed to decrease. These observations were further strengthened by reduction of expression of enzymes responsible for key metabolic functions including glycolysis and the TCA cycle, ATP generation by mitochondria and fatty acid β-oxidation (Davoodi et al., 2019). Under persistent IMD activation, the fly undergoes depleted fat reserves, hyperglycemia and impaired development (Davoodi et al., 2019). Further, IMD mutants showed hyperlipidemia, impaired insulin signaling and compromised glucose tolerance. Following these observations, the authors hypothesized that loss of metabolic regulation hindered the mounting of immune responses against microbial infections in the fly (Davoodi et al., 2019). Similarly, Martínez et al. has reported how TAG is diminished at the tissue level when the Toll signaling pathway is activated in the larval fat body of *Drosophila* (Martínez et al., 2020). The study also describes how enzymes of the Kennedy pathway, responsible for phosphatidylcholine and phosphatidylethanolamine homeostasis were increased upon activation of Toll signaling. In addition, transmission electron microscopy observations depicted how Toll signaling activation resulted in expansion of the endoplasmic reticulum (ER) volume in fat body cells. These observations provide compelling evidence that the metabolic landscape is intimately associated with immune signaling (Martínez et al., 2020).

Eicosanoids are fatty acid derivatives that can act as immunomodulatory molecules. They are mostly oxygenated metabolites of three C20 polyunsaturated fatty acids including arachidonic acid (20:4n-6), dihomo-gamma-linolenic acid (20:3n-6) and eicosapentanoic acid (20:5n-3). Eicosanoids are composed of 3 major groups of metabolites: prostaglandins, lipoxygenase metabolites, and epoxyeicosatrienoic acids (Stillwell, 2016). In insects, eicosanoids are known to mediate phagocytosis, micro aggregation, nodulation and encapsulation of invading microbes and metazoans (Stanley and Miller, 2006; Stanley and Shapiro, 2007). Since mosquitoes are unable to synthesize C20 polyunsaturated fatty acids, they require these fatty acids from diets (Blomquist et al., 1991; Sushchik et al., 2013).

All three groups of eicosanoid metabolites are found in mosquitoes (Petzel and Stanley-Samuelson, 1992; Ramirez et al., 2015; Xu et al., 2015). In *Anopheles gambiae* lipoxin A_4_ was found to be induced against the invasion of *Plasmodium* ookinetes in the midgut (Ramirez et al., 2015). The role of eicosanoids in mosquitoes against virus infection has only been reported in C6/36 cells (*Ae. albopictus* cells). Prostaglandin A_1_ was found to inhibit the replication of vesicular stomatitis virus in a dose-dependent manner (Burlandy et al., 2004). The role of eicosanoid metabolites in DENV infection of mosquitoes is still unknown. Chotiwan et al, 2018 observed that Prostaglandin A2 and D2 were upregulated in DENV2 infected *Ae. aegypti* midguts during early replication time points. Interestingly, DENV infection in human (Huh7) and dendritic cells induced the expression of cyclooxygenase-2 (COX-2), the enzyme that produces prostaglandin E_2_ (Wu et al., 2009; Lin et al., 2017). The production of prostaglandin E_2_ in infected cells was also enhanced and promoted migration of DENV infected dendritic cells from the upper to the lower chamber in culture (Wu et al., 2009). Mice that were treated with COX-2 inhibitor were protected from DENV infection (Lin et al., 2017). The role of prostaglandins in DENV infection in the mosquito remains to be investigated.

Autophagy is a cellular mechanism that removes unwanted debris and damaged organelles from a system. This process facilitates recycling of material as well as regeneration of newer cells. Another function of autophagy is intracellular pathogen clearance (Deretic and Levine, 2009). In insects, autophagy is important during metamorphosis, development, response to starvation as well as defense against pathogens (Tian et al., 2013; Romanelli et al., 2016; Tettamanti and Casartelli, 2019). However, viruses like DENV can seize this cellular mechanism to boost replication. Although the core mechanism is not well understood, experimental data reveals that autophagy induced by DENV modifies cellular lipid metabolism (Heaton et al., 2010).

Immunometabolism is a rapidly evolving discipline that investigates the relationship between metabolic homeostasis and immunity during infection. There is ample evidence to strengthen the argument of metabolic pathways being closely associated with cellular immune signaling pathways. However, immunometabolism is not well explored in arboviral vectors. There is a necessity to understand the mechanisms underlying the crosstalk between immune responses and cellular metabolic homeostasis. Such studies would provide a better understanding of the choke points that can be employed in pathogen blocking and vector control.

## 4 Metabolic processes are associated with senescence in mosquitoes

The normal life span of a wild mosquito can vary from approximately 10 to 60 days. Males have a shorter life span of nearly 10 days while females live longer for approximately 60 days (Ehrlich, 2022). However, these periods critically depend on environmental conditions such as temperature, humidity, and the availability of blood meals for females. As with any other organism, aging affects multiple physiological processes in mosquitoes. Digestion, mating, reproduction, flight and immunity are among some of the traits that are altered due to aging (Edman, 1970; Christensen et al., 1986; Hillyer et al., 2004; King and Hillyer, 2013; Sawadogo et al., 2013). In addition, aging can have a critical impact on the vectorial capacity of a mosquito. Any pathogen that is vectored by a mosquito needs to complete an extrinsic incubation period (EIP) prior to being transmitted (Cook et al., 2008). This EIP allows the pathogen to amplify within the vector. The inability of a mosquito to survive until the pathogen completes the EIP, renders a discontinuation in the transmission cycle. It is also important to note that with aging, immune responses of the vector might weaken thereby influencing vector competence (Boëte and Koella, 2003). Besides these alterations, metabolism of the mosquito is also prone to change since it is closely associated with the physiology of the organism. Discussed here are some of the key studies based on mosquito development, aging, and related metabolism.

### 4.1 Fatty acid synthesis

As discussed in this review, fatty acids are a group of vital lipids in mosquitoes serving as structural components in cellular membranes, energy homeostasis, signaling, innate immunity and reproduction. Fatty acid synthesis is conducted by a multifunctional enzyme complex called the fatty acid synthase complex (FAS) (Maier et al., 2008). In *Ae. aegypti* several paralogues of FAS were found (Chotiwan et al., 2022). The study investigated the dynamic expression of FAS genes in relation to developmental stages. Larval and pupal stages showed negligible FAS expression in comparison to adult stages (Chotiwan et al., 2022). This is consistent with the fact that larvae and pupae utilize maternal lipids deposited in the eggs and do not need to synthesize fatty acids in early life stages. All FAS genes except one isoform were highly expressed in adult male mosquitoes in comparison to other life stages. Since male mosquitoes do not feed on blood, they solely depend on nutrients taken up in a plant meal (nectar) and need FAS function to fulfil their lipid requirements (Chotiwan et al., 2022).

### 4.2 Glycogenesis and lipogenesis

Sugars obtained in the diet of mosquitoes are partially hydrolyzed by enzymes in the saliva when stored in the crop. During this temporal storage, salivary enzymes partially hydrolyze the ingested sugars to produce hexoses. Clements describes how these end products are utilized in the synthesis of glycogen (glycogenesis), fatty acids and triglycerides (lipogenesis) in proportions that are species and life stage specific (Clements, 1992). Several studies have also extensively investigated adult energy metabolism in multiple mosquito species and reported age specific trends of glycogenesis and lipogenesis (Briegel, 1990; Briegel and Timmermann, 2001; Ziegler and Ibrahim, 2001). Briegel et al., and Briegel and Timmermann investigated the accumulation of glycogen during the first week and lipids during the first two weeks in the adult life stages of *Ae. aegypti* and *Ae. albopictus* mosquitoes (Briegel, 1990; Briegel and Timmermann, 2001). In similar studies in *Culex tarsalis*, carbohydrates and lipids followed the same trend as in other mosquito species. However, lipid synthesis was more rapid than carbohydrate synthesis in *C. tarsalis* (Gray and Bradley, 2003). Further, *C. tarsalis* mosquitoes exhibited higher lipid storage trends in young adults in comparison to *Ae. aegypti* mosquitoes. These trends could be accounting for the autogenous potential of the Culex species which imply that the adults do not need a blood meal to lay eggs but can utilize lipid reserves (Gray and Bradley, 2003).

### 4.3 Glutathione Metabolism

Glutathione (GSH) is an important molecule for insects. This enzyme plays a critical role in a number of biosynthetic and detoxification reactions (Forman et al., 2009). Glutathione transferases (GST) are also important enzymes that play a role in detoxification of substances that can be both endogenous or xenobiotic. In insects, these enzymes are known to play a role in insecticide resistance (Enayati et al., 2005). Besides these functions, studies also discuss the alteration of GSH during aging of *Ae. aegypti* mosquitoes (Hazelton and Lang, 1983). The GSH biosynthesis rate was observed to be distinctly reduced in aging adult mosquitoes. Further, a considerable decrease in biosynthetic rates were observed during senescence of the mosquitoes (Hazelton and Lang, 1983). Impaired GSH biosynthesis leading to low levels of GSH implies that regular cellular functions of GSH such as detoxification of peroxides and xenobiotics will be impaired ultimately leading to tissue damage and death.

Vector control programs take the average age of a mosquito population as a crucial determinant of vectorial capacity and potential of disease transmission (Johnson et al., 2020). This makes mosquito age grading important in vector control. The technique in current use for age grading is the Detinova parity method that assesses the age of a female mosquito by taking the changes in ovary appearance into account (Gray et al., 2022). In addition, novel tools like surface – enhanced Raman spectroscopy (SERS) is currently being studied as a potential mosquito age grading technique (Wang D. et al., 2022). In a study by Wang et al., where age of a mosquito is determined by both SERS and Infrared spectroscopy states that key biological molecules are altered in a mosquito with age which subsequently alter the spectra obtained by either of the mentioned methods (Wang D. et al., 2022). However, in the field, there is a lack of capacity to accurately determine the age of a mosquito caught in the wild. Often the techniques used are impractical or unreliable (Johnson et al., 2020). Therefore, understanding metabolic changes occurring with aging in mosquitoes can be used to develop biomarker point-of-use tests for age grading as well as vector control (Iovinella et al., 2015).

## 5 Virus infection modulates metabolism in the mosquito

### 5.1 Barriers to infection

When a mosquito takes a viremic blood meal, the virus particles have to pass through several physical barriers in the mosquito in order to establish a successful infection, disseminate through the mosquito and be transmitted to a human host (Bosio et al., 2000). Infection of the midgut epithelium is the first barrier to infection. Presence of DENV, serotype 2 (DENV2) in the midgut tissue can be detected with the 3H5 monoclonal antibody as early as 2 days after the infectious blood meal was taken (Salazar et al., 2007). Staining at this early stage shows infected foci, indicating that the infection spreads laterally from the initial infected epithelial cells to the neighboring cells and eventually throughout the midgut (Salazar et al., 2007).

Upon successful infection of midgut epithelial cells, the virus must pass through the midgut escape barrier and continue to replicate in other tissues. Studies using electron-microscopy have shown that flaviviruses such as WNV and St. Louis encephalitis virus, escape from the midgut to the secondary tissues by passing through the basal lamina, the layer of extracellular matrix surrounding the midgut (Whitfield et al., 1973; Girard et al., 2005). Interestingly, a second non-infectious blood meal ingested by the mosquito enhances viral escape due to micro-perforations in the mid-gut (Armstrong et al., 2020) A study on DENV2 tropism in *Ae. aegypti* detected viral antigen in the trachea from the abdominal areas, suggesting that the trachea may also serve as an escape route for the virus from the midgut (Salazar et al., 2007). Following escape of the midgut barrier, the virus must replicate and amplify the infection in secondary tissues. Each of these tissues presents infection and escape barriers. A study has shown that DENV2 replicates in the fat body, hemocytes, nerve tissues, ommatidia of the compound eyes, esophagus, hindgut, cardia, trachea and Malpighian tubules (Salazar et al., 2007). Unlike WNV, DENV2 was not found to infect muscles (Girard et al., 2005; Salazar et al., 2007). Efficacious pass-through these barriers allows the virus to infect the salivary glands where the virus can be shed in the saliva when the next blood meal is taken to transmit to another host (Bosio et al., 2000).

### 5.2 Infection induced membrane rearrangements

In order for arboviruses to better survive in nature, they must evolve to survive in the invertebrate vector as well as the vertebrate host (Rückert and Ebel, 2018). Flaviviruses infect and rearrange the membrane architecture in their arthropod host cells like that observed in infected human cells (Girard et al., 2005; Welsch et al., 2009; Gillespie et al., 2010; Offerdahl et al., 2012; Junjhon et al., 2014; da Encarnação Sá-Guimarães et al., 2021; Mazeaud et al., 2021). Interestingly, these virus-induced membrane structures are morphologically and functionally conserved between these evolutionary distant hosts. These structures are summarized in **Table 1**. They are mostly endoplasmic reticulum (ER)-derived and include i) vesicles (Ve), the circular vesicular structures that house the viral replication complex, ii) vesicle packet (Vp), the larger vesicles that surround Ve, iii) convoluted membranes (CM), the site of viral protein translation and processing, and iv) tubular structures (T) with unknown function. In C6/36 cells infected with DENV2, Junjhon et al. observed Vp, Ve and T, with the number of Ve increasing with viral RNA copy number indicating a linear correlation between membrane structures and viral RNA replication (Junjhon et al., 2014). However, in contrast to DENV2 infected human Huh7 cells, CM were not found in infected C6/36 cells (Junjhon et al., 2014).

**Table 1:** Membrane rearrangements of host cells induced by virus infection.

Vesicle Type	Description	Virus	Cell Type / Organism
Convoluted Membranes (CM) 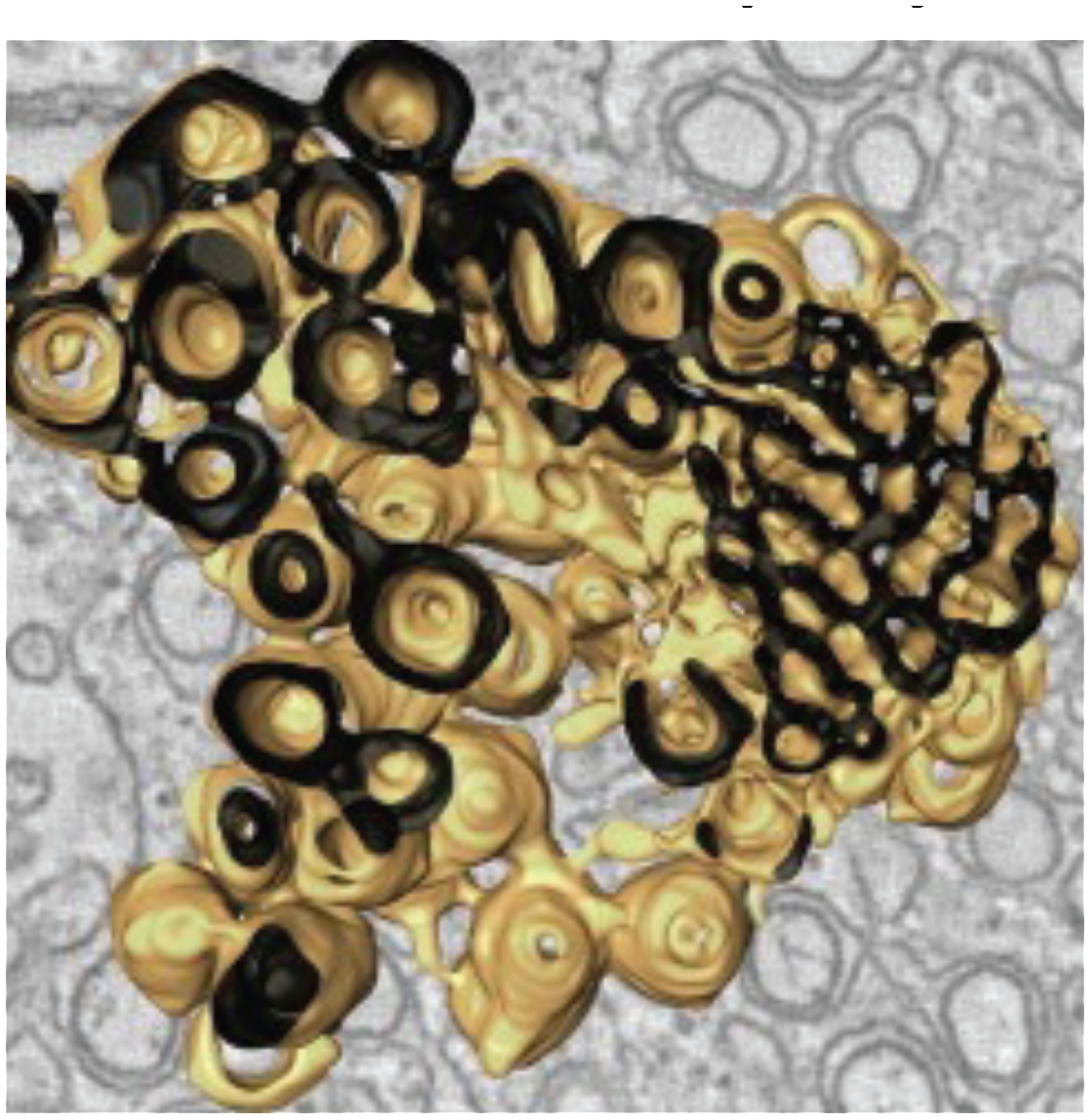 (Welsch et al., 2009)	• ER derived complex reticular network of membranes • Enriched in viral protease NS3 and co-factor NS2B • Presumed site for viral protein translation and polyprotein processing	DENV WNV TBEV LGTV	***Mammalian*** Vero cells Huh7 cells ***Arthropod*** Tick ISE6 cells Mosquito C6/36 cells Mosquito (Aedes aegypti) Salivary glands
Vesicles (Ve) / Vesicle packets (Vp) 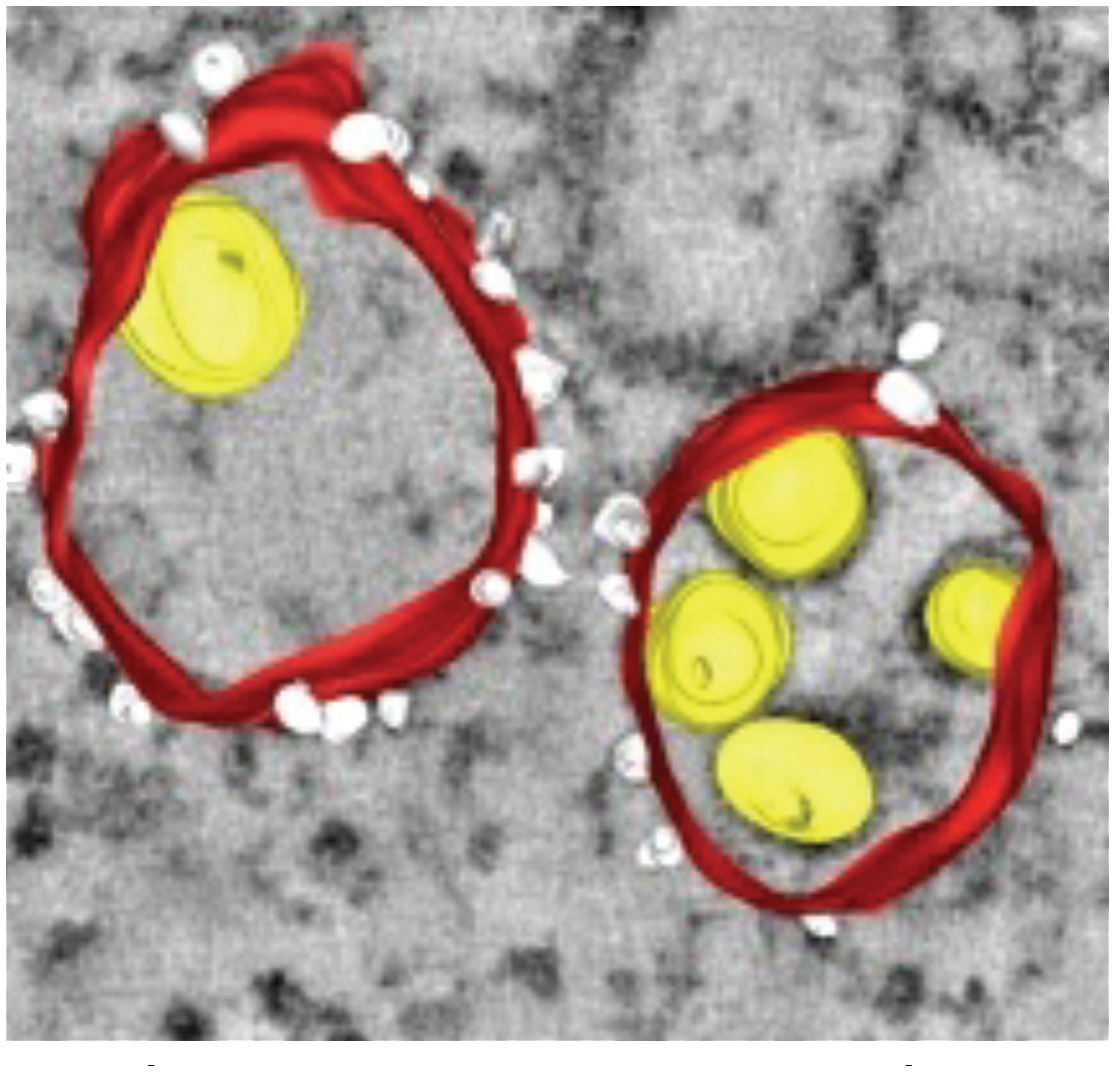 (Gillespie et al., 2010)	• Vesicle packets (Vp) are ER derived and contain invaginations of smaller internal vesicles (Ve) with kneck-like pores open to the cytoplasm • The presence of viral replicase proteins and dsRNA suggest this is the site of viral RNA replication	DENV WNV TBEV LGTV	***Mammalian*** Vero cells Huh7 cells ***Arthropod*** Tick ISE6 cells Mosquito C6/36 cells Mosquito (Aedes aegypti) Salivary glands
Packets of virus particles 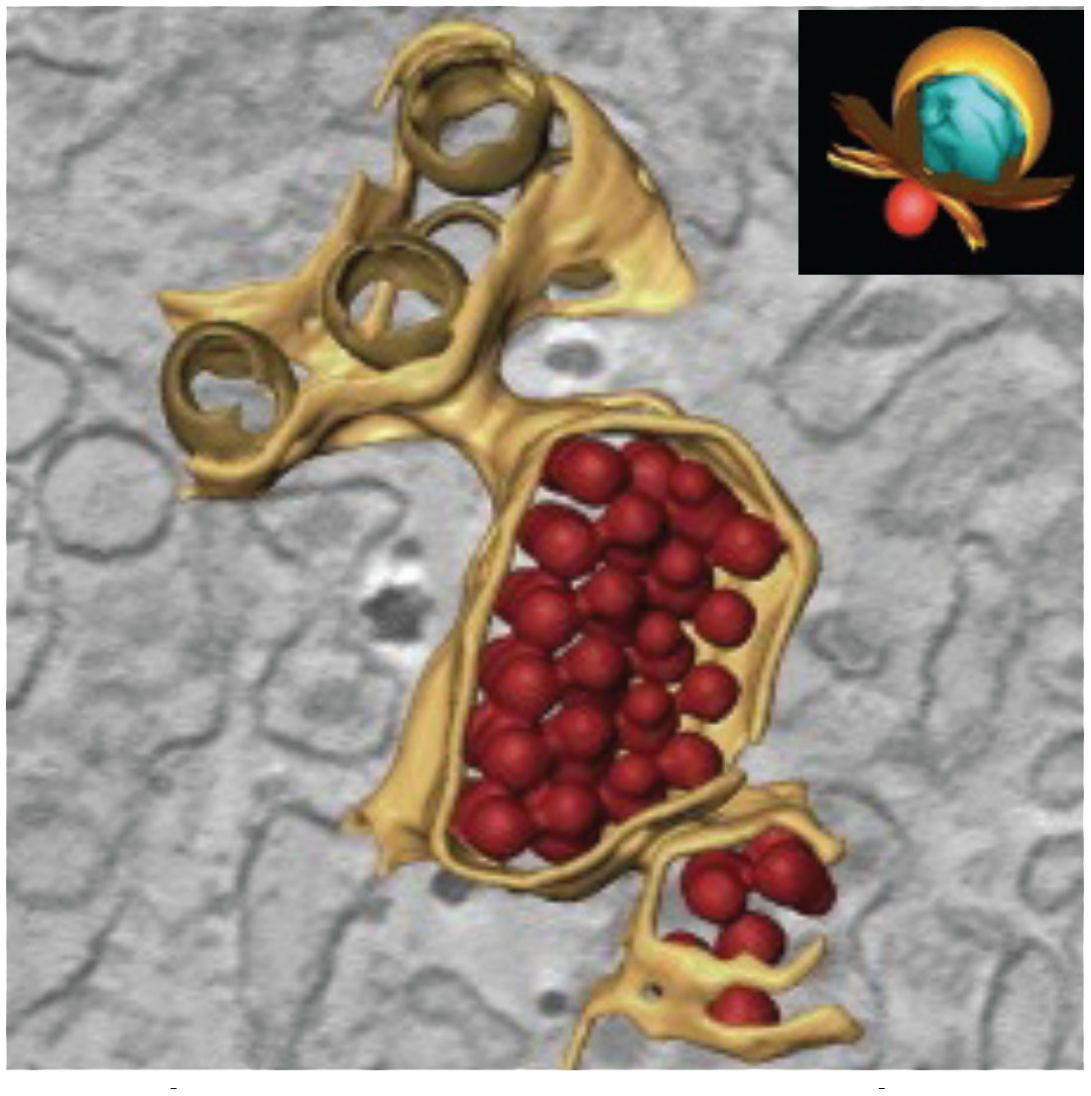 (Welsch et al., 2009)	• Vp/Ve (brown) are connected through membranous knecks to packets of newly assembled virus particles (red) • The inset shows how the Ve housing the replication complex (blue) is juxtaposed to the site of virus assembly	DENV WNV TBEV LGTV	***Mammalian*** Vero cells Huh7 cells ***Arthropod*** Tick ISE6 cells Mosquito C6/36 cells Mosquito (Aedes aegypti) Salivary glands
Tubular Structures 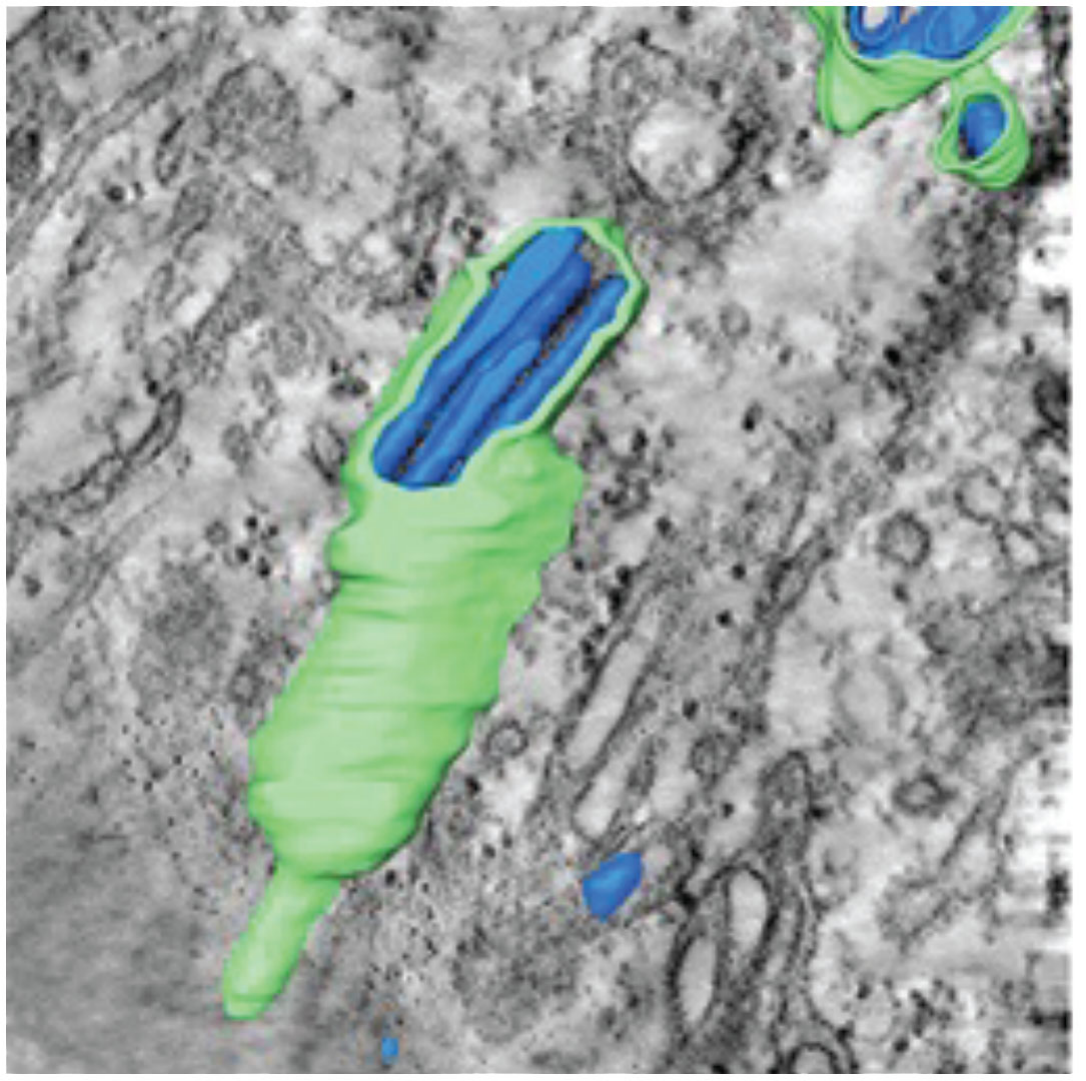 (Offerdahl et al., 2012)	• Fascile-like bundles of multiple tubules wrapped in a single membranous sheath • Single tube structures have been observed in both mosquito and tick cells but these sheaths are only observed in tick cells persistently infected with LGTV	LGTV	***Arthropod*** Persistently infected Tick ISE6 cells

Similar vesicular structures were observed in mosquito cell lines infected with other flaviviruses. Electron micrographs revealed the induction of similar vesicular structures in *Ae. albopictus* cells infected with Kunjin virus and *Ae. aegypti* cells infected with yellow fever virus (Ishak et al., 1988). These membrane-rearrangements were also observed in WNV infected *Culex quinquefasciatus* mosquito tissues (Girard et al., 2005). The above-mentioned vesicle structures were observed in the midgut epithelium, midgut muscle and salivary gland tissues indicating that this specific membrane architecture was universally induced in both human and mosquito hosts in response to infection with most flaviviruses. In addition to these discussed structures, DENV infection of C6/36 cells was reported to produce exosomes that could infect naive C6/36 cells. Based on these observations, the authors suggested that virus induced exosomes have infectious potential and supports viral dissemination in C6/36 cells (Reyes-Ruiz et al., 2019). These studies on membrane architecture, together with the studies on lipid composition of infected cells, suggest that in addition to rearranging cellular membrane architecture during infection, existing membrane lipids are reorganized (Vial et al., 2020) as well as additional lipids (such as GPs and sphingolipids, SPs) are synthesized and incorporated into these membranes to promote expansion of membrane mass. Lipids with unsaturated fatty acyl chains and cone-shaped lipids such as PE, lysoGPs and ceramides (Cer) were increased likely to provide curvature and membrane-bending capabilities to facilitate the specific architecture required. Essentially, there is a concerted effort (by viral gene products) to alter both lipid metabolism and cellular membrane architecture to acquire an intracellular environment conducive to viral replication (Roosendaal et al., 2006; Miller et al., 2007; Perera and Kuhn, 2008; Perera et al., 2012).

### 5.3 Lipid metabolism and its impact on viral infection

Several studies to date have highlighted the impact of viral infection on the mosquito metabolic landscape (**Table 2**) (Perera et al., 2012; Melo et al., 2016). The initial study to utilize metabolomics to profile the metabolic landscape was carried out by Perera et al, on C6/36 *Ae. Albopictus* cells infected with DENV2. The study highlighted that ~15% of the metabolome was altered by infections of these cells. Lipid changes included those that had the capacity to alter membrane curvature and destabilize architecture, fluidity, and permeability. Specifically, GPs with smaller head groups such as phosphatidylethanolamine (PE) and cone-shaped (ie: ceramide) and inverse-cone shaped lipids (ie: lysophospholipids) were elevated in infected cells, specifically in membranes enriched in the replication complex. They also observed changes in SPs and glycerolipid intermediates such as monoacylglycerols (MAG) and DAG which are bioactive signaling molecules that participate in membrane fusion, fission, and trafficking and capable of enhancing a conducive environment for viral replication (Perera et al., 2012). These studies also demonstrated that like observations in human cells, *de novo* fatty acid biosynthesis *via* the enzyme, FAS, was important for viral replication in mosquito cells. Subsequently, a metabolomics study on Zika virus infected C6/36 cells identified 13 similar lipid species as specific biomarkers of infection (Melo et al., 2016). These included several species of SPs, GPs such as phosphatidylcholine (PC), phosphatodylserine (PS) and PE, as well as the bioactive intermediates such as DAG.

**Table 2:** 

Lipid Classes	Roles in mosquitoes1	Roles in virus infection2
**Glycerophospholipids** **(GP)**	Aggregated along the intestinal gastric caeca aiding food ingestion	Several GP species were elevated in DENV and ZIKV infected C6/36 and Aag2 cells. Some were enriched at the replication complex in cells.
Phosphatidylcholine (PC)	A major phospholipid in mosquito cells (30-40%), neutral, cylindrical lipid, forms planar bilayers	Elevated in DENV and ZIKV infected C6/36 cells*. de novo* biosynthesis was blocked but existing PCs were reorganized to the replication complex of DENV infected *Aag*2 cells. Elevated at peak viral replication in DENV infected midguts in *Ae. aegypti* mosquitoes.
Phosphatidylethanolamine (PE)	A major phospholipid in mosquito cells (26-45%), inverted cone-shaped lipid with a small, polar head group. Induces negative membrane curvature.	Elevated in the replication complex in DENV infected C6/36 cells, but in the form of lysophospholipids. Similar observations in DENV infected *Aag*2 cells. Increased in ZIKV infected C6/36 cells. PE associated with viral particles are involved in viral entry. Elevated at peak viral replication in DENV infected midguts in *Ae. aegypti* mosquitoes.
Phosphatidylserine (PS)	Similar percentage in mosquito cells as PI (~6.6%). Anionic lipid, enriched in the inner leaflet of mammalian plasma membranes. Exposed on the outer leaflet during apoptosis.	Enriched in viral envelops. Facilitates viral entry. Increased in ZIKV infected C6/36 cells and DENV infected *Aag*2 cells, decreased in DENV infected C6/36 cells. Elevated at peak viral replication in DENV infected midguts in *Ae. aegypti* mosquitoes.
Phosphatidylinositol (PI)	Similar percentage in mosquito cells as PS (~6.7%). Anionic lipid.	Observations are limited. Observed as lysophosphotidylinositol and increased in DENV infected *Aag*2 cells. Elevated at peak and late viral replication in DENV infected midguts in *Ae. aegypti* mosquitoes. Increased in DENV infected *Ae. aegypti* whole mosquitoes.
Phosphatidylglycerol (PG)	Synthesized in the mitochondria. A key intermediate in the biosynthesis of cardiolipin	Observations are limited. PGs Elevated in infected midguts at early and mid-time points. PGs with shorter fatty acid chains elevated at late time points, and PGs with longer fatty acid chains elevated at early time points post-infection in whole mosquitoes.
Phosphatidic acid (PA)	Minor phospholipid in mosquito cells (~1%). Anionic lipid. Precursor of more complex lipids. Roles in cell signaling and lipid-gated ion channels. Induces membrane curvature.	Observations are limited. Elevated in DENV infected replication complex membranes in C6/36 cells. Elevated in DENV infected midguts at early, mid and late time points in *Ae. aegypti* mosquitoes.
Lysophospholipids (LPL)	Minor phospholipid in *Ae. aegypti c*ells, mostly abundant in larvae of mosquitoes. Inverse-cone-shaped lipid, induces positive membrane curvature, signals through GPCRs	Elevated in DENV infected C6/36 and *Aag*2 cells and enriched at the DENV replication complex. Decreased throughout infection in DENV infected midguts in in *Ae. aegypti* mosquitoes. Elevated during early DENV infection and decreased at later time points in whole mosquitoes.
**Sphingolipids (SP)**	Regulation of energy homeostasis, fat body metabolism, phototransduction, brain development and behavior in Drosophila. Less studied in the mosquito.	Several SPs are elevated in DENV infected cells and mosquitoes. Ceramides specifically elevated in infected cells and enriched at the replication complex of C6/36 cells. Ceramides also significantly elevated in the DENV2 infected Ae. aegypti midguts. Sphingomyelins elevated in C6/36 cells following infection with DENVs. Unchanged in DENV infected midguts in *Ae. aegypti* mosquitoes. Not significantly observed in other studies.
**Glycerolipids (GLs)**	Important lipid source for energy metabolism of the mosquito. Glycerolipids form the core of lipid droplets in the mosquito fat body	Several glycerolipids were observed to be increased in DENV infected *Ae.aegypti* mid guts during early time points post infection. Some glycerolipids were reduced in replication complex membranes isolated from DENV infected C6/36 cells.
Monoacylglycerols (MAGs)	Bioactive signaling molecules that participate in membrane fusion, fission, and trafficking. Critical effectors of energy metabolism in insects.	Capable of enhancing a conducive environment for viral replication.
Diacylglycerols (DAGs)	DAGs are second messengers that regulate cell proliferation, mitochondrial physiology, apoptosis and survival. Class of lipids forming the lipophorins in many insects. Act as Intermediates in GP synthesis.	Significantly elevated in the DENV2 infected *Ae. aegypti* midguts during early time points post infection. Have been identified as biomarkers in ZIKV infected C6/36 cells.
Triacylglycerols (TAGs)	Triglycerides are the major source of stored lipids in mosquitoes. Important for energy metabolism, oogenesis and diapause of the insect. Also important for the development of the mosquito. These are known to increase body size and fecundity in adult mosquitoes.	High levels of TAGs were detected in DENV infected *Ae. aegypti* mosquito midguts on day 3 and 7 post infection. It is possible that TAGs are transported from storage sources to support lipid demand in other tissues during infection.
**Cholesterol**	Cholesterol is a precursor for steroid hormone, 20- hydroxyecdydone (20E) which regulates blood meal digestion and egg development in female mosquitoes and maintains sperm integrity in male mosquitoes.	Cholesterol is identified to be essential for flavivirus entry, replication and assembly in human cells. Modulation of endogenous cholesterol biosynthesis or exogenous cholesterol uptake alters DENV replication as well as WNV in human cells. RNAi and inhibitors against Sterol Carrier Protein-2 ( SCP-2) altered cellular cholesterol distribution and reduced DENV titers in *Aag*2 cells. RNAi against Neiman Pick Type C1 (NPC1) protein reduced DENV infection in the midgut of lab and field *Ae. aegypti* mosquitoes*. Wolbachia* perturbs cholesterol trafficking and inhibits DENV in *Ae. aegypti* cells.
**Fatty acids**	Precursor for synthesizing eicosanoids, pheromones, GPs and wax, bioactive molecules in cellular signaling, Essential for numbers and viability of eggs, distributed along the sides of the larval body, associated with muscle to provide energy for locomotion.	Fatty acids and derivatives were elevated levels in DENV infected *Ae. Aegypti* midguts. Inhibition of AaFAS reduces DENV2 infection in midgut of *Ae. aegypti*.
Polyunsaturated fatty acids	Proper functioning of innate immunity, developmental processes and flight in adult stage. Includes eicosanoids such as prostaglandins, lipoxygenase metabolites and epoxyeicosatrienoic acids that mediate phagocytosis, micro aggregation, nodulation and encapsulation of invading microbes and metazoans.	Lipoxin A4 was found to be induced against the invasion of Plasmodium ookinetes in the midgut. Prostaglandin A1 inhibits vesicular stomatitis virus replication in C6/36 cells. Prostaglandin A2 and D2 and thromboxane were upregulated in DENV2 infected *Ae. aegypti* midguts, but the role is unknown.
Acylcarnitines	Acylcarnitines play a critical regulatory role in generating energy from lipid stores. Binding of carnitines to fatty acyl-CoA molecules via the activity of carnitine palmitoyl transferase (CPT) generates acylcarnitines that are then shuttled to the mitochondrial matrix for energy generation through β-oxidation. Acyl-carnitines were observed to be decreasing during early diapause of *Ae. albopictus* mosquitoes suggesting that carnitine shuttle is suppressed in early diapause contributing to lipid conservation via reduced β-oxidation	Significantly elevated levels of 26 acylcarnitines were observed in DENV2 infected *Ae. Aegypti* midguts. Only one acylcarnitine was detected to be decreased in abundance.25 out of 26 increased acylcarnitines had medium length fatty acyl chains of 4-12 carbons suggestive of incomplete β-oxidation. *Wolbachia (wMel)* infected *Ae. Aegypti* cells (*Aag*2) showed significantly low levels of acylcarnitines in comparison to *Wolbachia* free cells. However, infection with DENV-1, ZIKV (African) or ZIKV (Asian) strains caused an increase in acylcarnitine levels in infected Aag2 cells in comparison to uninfected cells. Contrastingly, superinfection with *Wolbachia* and virus lead to drastic reduction in majority of acylcarnitines proposing that Wolbachia is modulating acylcarnitines that in turn affect virus replication.

1 References: [Clayton, 1964; Townsend et al., 1972; McMeans et al., 1975; Butters and Hughes, 1981; Knabb et al., 1986; Ishak et al., 1988; Kler et al., 1991; Jenkins et al., 1992; Pennington et al., 1996; Ziegler, 1997; Ziegler and Ibrahim, 2001; Pennington and Wells, 2002; Renkonen et al., 2002; Sanders et al., 2003; Attardo et al., 2005; Stanley and Miller, 2006; Brasaemle, 2007; El-Bacha et al., 2007; Koves et al., 2008; van Meer et al., 2008; Farese and Walther, 2009; Guo et al., 2009; Stone et al., 2009; Arrese and Soulages, 2010; Bakermans et al., 2013; Holthuis and Menon, 2014; Fontaine et al., 2015; Ramirez et al., 2015; Roy et al., 2015; Abdul Rahim et al., 2018; Ekoka et al., 2021; Silva et al., 2021].

2 References: [Clayton et al., 1964; Dadd and Kleinjan, 1979; Dadd et al., 1987; Atella and Shahabuddin, 2002; Burlandy et al., 2004; Stanley and Miller, 2006; Mackenzie et al., 2007; Nene et al., 2007; Vyazunova and Lan, 2010; Alabaster et al., 2011; Liu, 2012; Perera et al., 2012; Carro and Damonte, 2013; Guan et al., 2013; Nasheri et al., 2013; Soto-Acosta et al., 2013; Jupatanakul et al., 2014; Moller-Tank et al., 2014; Carnec et al., 2015; Fu et al., 2015; Ramirez et al., 2015; Richard et al., 2015; Melo et al., 2016; Geoghegan et al., 2017; Chotiwan et al., 2018; Vial et al., 2020; Liu et al., 2021].

Interestingly, in an elegant study using isotopically labelled precursors, Vial et al, determined that at early time points, *de novo* biosynthesis of aminophospholipids such as PC and PE was actively blocked by DENV infection of *Ae. Aegypti* (*Aag*2) cells and instead that existing amino PLs were reorganized into replication complexes (Vial et al., 2020). These studies also demonstrated that in *Ae. Aegypti* mosquitoes, the rate-limiting enzyme that catalyzes aminoPL biosynthesis, acylglycerolphosphate acyltransferase (AGPAT), was decreased by DENV infection further supporting the hypothesis that aminoPL reorganization rather than *de novo* biosynthesis was activated during early infection (Vial et al., 2019)

In the adult mosquito, Chotiwan et al. demonstrated how lipid metabolism was temporally altered in infected *Ae. aegypti* mosquito midguts (the first site of viral replication) during infection with DENV2. In this study, GPs, SPs and fatty acids were significantly elevated and correlated temporally with the development of viral replication in the midgut (Chotiwan et al., 2018). GPs in insects play a critical role in tolerance to environmental changes (Guan et al., 2013). Elevation of glycerolipid intermediates suggested that resources were diverted from energy storage to biosynthesis during infection. Increased acyl-carnitines signaled functional disruptions in mitochondrial activities and energy production. Therefore, this study highlighted that significant metabolic perturbations occurred at early stages of viral replication in the mosquito.

#### 5.3.1 Fatty acids and derivatives

Fatty acids are synthesized *via* the *de novo* fatty acid biosynthesis pathway and are precursors that are incorporated into complex lipid molecules. When fatty acids are linked to coenzyme A, they become activated and can be incorporated into complex lipids such as GPs, SPs and glycolipids (GLs) that can serve as structural components in membranes as well as bioactive molecules in cellular signaling. As independent entities, fatty acids and derivatives also have roles in signaling, energy homeostasis and the immune response.

Numerous studies in both mammalian and mosquito systems have shown that *de novo* fatty acid biosynthesis *via* FAS activity is a critical function required to support viral replication (Ishak et al., 1988; Roosendaal et al., 2006; Martıń-Acebes et al., 2011; Offerdahl et al., 2012; Nasheri et al., 2013; Tonglunan et al., 2017; Reyes-Ruiz et al., 2019; Vial et al., 2020; Chu et al., 2021; Liu et al., 2021; Mazeaud et al., 2021) Chotiwan, et al., 2018 also showed that numerous putatively identified fatty acids and derivatives were elevated in DENV infected *Ae. Aegypti* midguts compared to controls. Species such as fatty amides, hydroxy fatty acids, fatty amines, glycosides, dicarboxylic acids, keto fatty acids, eicosanoids and leukotrienes were detected (Chotiwan et al., 2018). Eicosanoids are known to be players of immunity in insects (Stanley and Miller, 2006). Prostaglandin A2, prostaglandin D2 (PGD2), PGD2-dihydroxypropanylamine and thromboxane, eicosanoid subspecies, were elevated in DENV infected mosquito midguts (Chotiwan et al., 2018). These molecules are known to have a potential signaling function in *Drosophila* (Tortoriello et al., 2013). Unfortunately, unlike in mammalian systems, our current knowledge on these numerous bioactive molecules in the mosquito are limited to detection and quantification following exposure to virus infection. Future studies will need to elucidate the exact mechanisms of how these molecules might function to support or limit viral infection in the mosquito vector.

#### 5.3.2 Acyl-carnitines

Acyl-carnitines are esters of L-carnitine and fatty acids and belong to a large class of metabolites that are also identified as non-protein amino acids. These molecules act as intermediates that shuttle fatty acyl-CoA from the cytoplasm into the mitochondria for β-oxidation and energy production. These are known to be critical regulators of energy conservation in diapausing mosquitoes (Batz and Armbruster, 2018)High resolution liquid chromatography mass spectrometry analysis of DENV2 infected *Ae. aegypti* midguts have revealed that numerous acyl-carnitines were significantly increased following infection. Interestingly, many of these elevated molecules had medium chain fatty acids (Chotiwan et al., 2018). Medium length fatty acyl chains are generated due to incomplete β-oxidation resulting from mitochondrial overload (Koves et al., 2008). Two hypotheses were presented: i) accumulation of acyl-carnitines during viral infection could be caused by stalling of their transport into the mitochondria resulting in a blockage or inhibition of β-oxidation. This is observed in cells exposed to hypoxia as well as in mammalian systems during DENV infection (Knabb et al., 1986; Kler et al., 1991; El-Bacha et al., 2007; Bakermans et al., 2013; Fontaine et al., 2015). This scenario could result in lipid partitioning and diversion of fatty acyl-CoAs into complex lipids required for virus-induced membrane expansion, at the expense of fatty acid oxidation, ii) Alternately, the accumulation of medium chain length acyl-carnitines could be due to a bottleneck caused by a large proportion entering the mitochondria inducing mitochondrial overload. This results in only a proportion of the molecules being processed *via* β-oxidation. Future studies will need to explore the molecular mechanisms of these scenarios and determine how mitochondrial energetics relate to viral infection success.

#### 5.3.3 Sphingolipids

SP are critical for structural integrity of cellular membranes. However, they also play critical roles as bioactive signaling molecules involved in stimulating many processes in the cell (Hannun and Obeid, 2008). In insects, the best-known information on SPs is from studies in *Drosophila* (Acharya and Acharya, 2005). These studies have shown that SPs are involved in the regulation of energy homeostasis, fat body metabolism, phototransduction, brain development and behavior (Acharya et al., 2008; Dasgupta et al., 2009; Bauer, 2010; Kohyama-Koganeya et al., 2011; Kraut, 2011; Chen et al., 2016). The functions of SPs in *Ae.* species are less well studied (Townsend et al., 1972; Jenkin et al., 1975; McMeans et al., 1975). Studies by our group on *Ae. albopictus* (Perera et al., 2012) and *Ae. aegypti* cells and *Ae. aegypti* mosquitoes (Chotiwan et al., 2018) have revealed a significant perturbation of SPs during infection with dengue viruses with many molecular species elevated and required for infection. Specifically, Chotiwan et al. demonstrated that a central hub in the SP pathway that interconverted dihydroceramide to ceramide was required for the virus life cycle. Additional studies on the impact of *Wolbachia* on SP metabolism in *Ae. aegypti* are discussed below.

#### 5.3.4 Cholesterol

Cholesterol was shown to be essential for flavivirus entry, replication and assembly in human cells (Mackenzie et al., 2007; Lee et al., 2008; Rothwell et al., 2009; Carro and Damonte, 2013). Manipulation of cholesterol biosynthesis either by RNA interference (RNAi)-mediated gene silencing of cholesterol biosynthesis genes or using inhibitors of cholesterol biosynthesis enzymes such as lovastatin reduced DENV and WNV replication in human cells (Mackenzie et al., 2007; Rothwell et al., 2009). Intracellular availability of cholesterol was also shown to facilitate successful DENV replication in mosquito cells and mosquito vectors (Tree et al., 2019). Mosquitoes cannot synthesize cholesterol *de novo* and need to acquire cholesterol exogenously such as from the microbiome or from food (Clayton et al., 1964). As a result, mosquitoes rely on the processes for cellular absorption, trafficking and metabolism of cholesterol. Transcription and protein expression of host factors that are involved in cholesterol trafficking and homeostasis were increased upon DENV infection of *Aag*2 cells, indicating that these cellular factors were viral agonists (Fu et al., 2015). Sterol carrier protein-2 (SCP-2) is a cytosolic protein involved in cholesterol binding and transport in mammalian cells (Krebs and Lan, 2003; Vyazunova and Lan, 2010). Studies by Fu et. al., found that inhibition of SCP-2 using RNAi mediated gene silencing or the SCP-2 inhibitor (N-(4-{[4-(3,4-dichlorophenyl)-1,3-thiazol-2-yl]amino}phenyl)acetamide hydrobromide) altered the cellular distribution of free cholesterol and also significantly reduced DENV titers in *Aag*2 cells (Jupatanakul et al., 2014; Fu et al., 2015). Genome-wide transcriptomic analyses of *Ae. aegypti* (Liverpool strain) revealed that the transcripts of members in the lipid-binding protein gene families, the myeloid differentiation 2-related lipid recognition protein (ML) and Niemann Pick type C1 (NPC1) families, were increased upon DENV infection (Nene et al., 2007; Jupatanakul et al., 2014). These proteins function in cholesterol absorption, trafficking and metabolism in mosquitoes (Jupatanakul et al., 2014). Loss-of-function studies using RNAi mediated gene silencing of these genes reduced DENV infection in the midgut of both lab-adapted and field-derived strains of *Ae. aegypti* (Jupatanakul et al., 2014). Lastly, Geoghegan et. al., have shown that *Wolbachia*, an intracellular endosymbiotic bacterium, inhibited DENV in *Ae. aegypti* cells by perturbing cholesterol trafficking and causing accumulation of cholesterol in lipid droplets (Geoghegan et al., 2017). A compound, 2-hydroxyorioyl-β-cyclodextrin, that restores lysosomal cholesterol accumulation in Niemann-Pick type C disease rescued DENV replication in *Wolbachia*-infected mosquito cells (Liu, 2012; Geoghegan et al., 2017). In summary, these studies have shown the importance of cholesterol metabolism and intracellular trafficking, which play agonist roles facilitating DENV infection and replication in mosquito vectors.

#### 5.3.5 Lipid droplets and lipid reserves

Lipid droplets (LDs) are ER derived organelles that store neutral lipids like TAGs and sterol esters (Tauchi-Sato et al., 2002). They have a hydrophobic core consisting of these neutral lipids and are surrounded by a phospholipid monolayer associated with a specific repertoire of proteins (Brown, 2001; Bartz et al., 2007; Olzmann and Carvalho, 2019). These proteins have multiple functions and belong to numerous protein families such as enzymes involved in lipid synthesis (Kuerschner et al., 2008; Stone et al., 2009), lipolysis and membrane trafficking and proteins involved in maintaining structural integrity (Brasaemle, 2007; Guo et al., 2009). Recently, LDs were fully entitled as organelles with the primary function of lipid and energy homeostasis (Farese and Walther, 2009). LDs also serve the purpose of shielding the cell from toxic effects of excess lipids by compartmentalization of lipids. Mosquitoes store lipids acquired from the blood meal in lipid droplets (LDs). While LDs are found in almost all tissues in the mosquito, they are enriched in adipocytes in the fat body. These LDs can have dynamic sizes depending on the nutritional and metabolic status of the mosquito (Pinch et al., 2021). Lipids in LDs can be catabolized to generate energy *via* β-oxidation, provide building blocks for membrane biogenesis and bioactive molecules for signaling. Studies in *Drosophila* have shown that LDs are also players in intracellular protein metabolism (Cermelli et al., 2006; Farese and Walther, 2009).

TAGs accounts for the most common storage lipid in insects (Arrese and Soulages, 2010). In mosquitoes, TAGs from LDs and other tissues in the fat body are transported between tissues in the rest of the mosquito *via* lipophorins (Ford and Van Heusden, 1994; Van Heusden et al., 1997; Pennington and Wells, 2002). DAGs are lipolytic products of TAG and are believed to be the major class of transported lipids in most other insects. DAGs serve as intermediates in GP synthesis (van Meer et al., 2008; Sarri et al., 2011) and play a key role as a second messenger that regulates cell proliferation, mitochondrial physiology, apoptosis and survival (Mérida et al., 2008; Lin et al., 2014).

In addition to the above discussed functions, LDs are also known to have a relationship with activating Toll-like receptors (TLR), during DENV infection (Barletta et al., 2016). Barletta et al., have shown that LDs accumulated in *Aag*2 cells when challenged with DENV or Sindbis virus or a bacterial pathogen (Barletta et al., 2016). Further, LDs were observed to accumulate in midgut cells of mosquitoes as a response to bacterial or viral infection. Interestingly, this accumulation of LDs in the mosquito midgut cells occurred when the native microflora was proliferating after a blood meal (Barletta et al., 2016). As proposed by Barletta et al., buildup of LDs in infected mosquito cells and tissues is suggestive of an immune role played by LDs. Alternately, it could also serve as an energy reserve for the microflora. Additional research needs to be conducted to fully unravel the coupled relationship between insect immune responses and LDs.

Studies have reported increased numbers of LDs in dengue infected cells in response to the virus. This observation was seen in both mammalian cells (BHK, HepG2) and mosquito cells (C6/36 and Aag2) (Samsa et al., 2009). It was proposed that there is a possible crosstalk between viral replication and LD metabolism. The authors demonstrated that DENV replication was disrupted when LD formation was pharmacologically inhibited (Samsa et al., 2009). Concurrently, Heaton and Randall showed that DENV infection induced autophagy to regulate cellular lipid metabolism. This study observed an increase in the number of LDs but they were much smaller in size. The authors hypothesized that LD stored lipids like TAGs were being broken down to free fatty acids that could be shunted to increase cellular β-oxidation and generate more ATP required for virus replication (Heaton and Randall, 2010).

In summary, LDs as metabolic organelles play a critical role in mosquito lipid storage, transport, metabolism and energy homeostasis. Evidence suggest that they may also have a role in immunity that impacts both the microbiome and possibly pathogen transmission. While our knowledge of LDs has been gathered from studying these organelles in mammalian systems, there is a critical gap in our understanding of the molecular mechanisms that drive LD formation, activation and metabolism in relation to the needs or responses of the mosquito to environmental queues, the microbiome and pathogen infection.

## 6 Vector control, pathogen blocking and metabolism

As discussed above, metabolic homeostasis is important for proper biological functions of an organism. Every stimulus including environmental changes (temperature, humidity), exposure to insecticides, the microbiome including transinfected microbiota (*Wolbachia*) can alter the metabolic landscape. These altered metabolic landscapes can have significant impact on obligatory pathogens such as viruses that require resources of the host to carry out successful replication. In this section we discuss the metabolic impact of insecticides, insecticide resistance, the microbiome and biocontrol strategies such as *Wolbachia* on the mosquito and vector competence.

### 6.1 Insecticide resistance is integrated with metabolic changes in the mosquito

**Figure 3:**
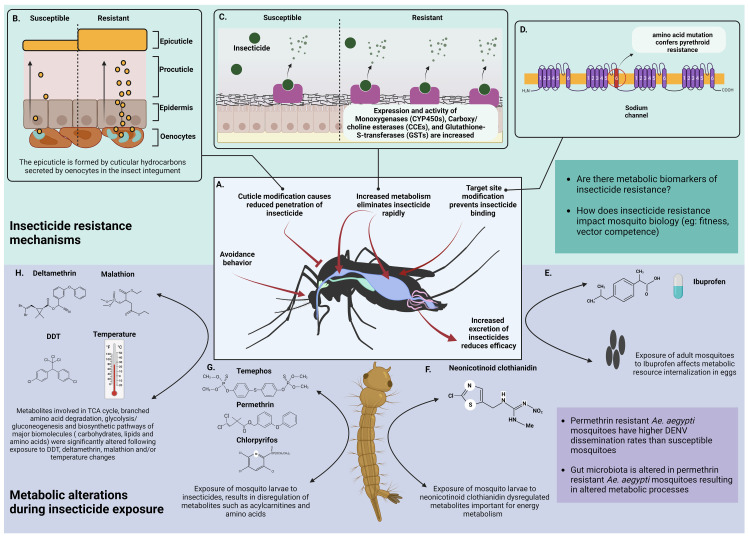
Metabolic basis of insecticide resistance. **(A)** General strategies of insecticide resistance shown by mosquitoes. **(B)** Resistance is acquired through modification of the integument by thickening the cuticle (image adapted from Bass et al., 2016) (Bass and Jones, 2016) **(C)** Insecticide resistant mosquitoes show higher expression and activity of esterases. These enzymes rapidly bind with incoming insecticide molecules and metabolize them thereby inhibiting their activity. The impact of the metabolites following insecticide degradation on mosquito metabolism is unknown. **(D)** Mosquitoes acquire insecticide resistance by mutating the targeted binding sites (Oppenoorth, 1984; Scott, 1990) **(E)** Direct exposure of adult mosquitoes or larvae to Ibuprofen (an ubiquitous surface water contaminant) had no direct effect but it dysregulated internalization of metabolic resources like amino acids, carbohydrates, polyols, phosphoric acid and ornithine in F1 progeny (Prud’homme et al., 2018) **(F)** *Culex pipiens* larvae exposed to varying concentrations of neonicotinoid clothianidin had differences in metabolites important for energy metabolism (acylcarnitines, GPs and biogenic amine abundance). Higher concentrations of clothianidin reduced acylcarnitines, GPs and biogenic amines after 24 hours of exposure. Low and medium concentrations of clothianidin reduced GPs and amines but increased acylcarnitines indicating that low pesticide doses increased energy requirements of exposed larvae (Russo et al., 2018) **(G)** Exposure of *Culex quinquefasciatus* larvae to Temephos, Permethrin and Chlorpyrifos dysregulated acylcarnitines and the amino acid arginine. Arginine levels were increased following exposure to Permethrin and Temephos whereas acylcarnitines responded differently to each insecticide (Martin-Park et al., 2017) **(H)** Major biological pathways are altered due to exposure of mosquitoes to insecticides such as DDT, deltamethrin and malathion and/or temperature (Singh et al., 2022) *Created with BioRender.com*.

Vector control has been critical in the prevention and control of several vector-borne diseases. The use of insecticides to kill or deter vectors has been the mainstay of vector control globally (van den Berg et al., 2021). Intense insecticide use has selected mechanisms of resistance that are now prevalent in malaria vectors such as *Anopheles gambiae*, *Anopheles sinensis*, and *Anopheles funestus* (Hancock et al., 2018), as well as arbovirus-vectors such as *Ae. aegypti* and *Ae. albopictus* (Moyes et al., 2017). Insecticide resistance mechanisms include decreased cuticular penetration of insecticides, increased enzyme metabolism, and decreased sensitivity of insecticide target sites (Oppenoorth, 1984; Scott, 1990). The mechanisms of metabolic resistance will be the focus of this section (**Figure 3**).

Metabolic resistance protects against all insecticides used in public health, including pyrethroids, organophosphates, carbamates, and organochlorines (Smith et al., 2016) Insecticide metabolism is conducted by three enzyme families, cytochrome P450 monooxygenases (P450), glutathione transferases (GST), and carboxy/cholinesterases (CCE) (Strode et al., 2008; Feyereisen, 2012). Enhanced metabolism in resistant insects can be caused by gene over-expression (cis/trans regulation and gene amplification) or allelic variation of members of these enzyme families.

Because of the complexity of the enzyme family systems and the difficulty in purifying these enzymes (e.g., substrate overlap, instability, yields), understanding the mechanisms of resistance have proved difficult. The development of molecular and bioinformatics tools enabled the identification of genes and associated regulatory processes in resistant insects resulting in significant progress over the last decade. Several studies have investigated the differential gene expression of CYPs, GSTs, and CCEs in resistant *Anopheles, Culex*, and *Aedes* mosquitos (Smith et al., 2016; Moyes et al., 2017; Vontas et al., 2020). However, relatively few detoxification enzymes have been examined *in vitro* to validate their ability to metabolize insecticides. For example, CYP6P3, CYP6M2, and GSTE2 in *An. gambiae* have shown to metabolize pyrethroids, DDT, and bendiocarb (Müller et al., 2008; Stevenson et al., 2011; Mitchell et al., 2012; Yunta et al., 2019). Pyrethroids are metabolized by CYP6P9a and CYP6P9b in *An. funestus* (Riveron et al., 2014) and by CYP9M6, CYP6BB2, CYP9J24, CYP9J26, CYP6J28 and CYP9J32 in *Ae. aegypti* (Mitchell et al., 2012; Kasai et al., 2014). The role of these enzymes in pyrethroid resistance has been validated *in vivo* utilizing heterologous expression in *Drosophila* (Pavlidi et al., 2012; Edi et al., 2014; Reid et al., 2014; Riveron et al., 2014; Ibrahim et al., 2015) or RNA interference (RNAi) technologies in *Ae. albopictus* (Xu et al., 2018). A GAL4/UAS expression system was recently developed in *An. gambiae* to confirm *in vivo* that overexpression of GSTE2 conferred organophosphate and organochlorine resistance, CYP6P3 conferred pyrethroid and carbamate resistance, and CYP6M2 conferred pyrethroid resistance when overexpressed in the same tissues (Adolfi et al., 2019).

Metabolic technologies have recently been utilized to investigate the mosquito’s reaction to insecticides and to find metabolic routes of insecticide detoxification. For example, Prud’homme et al used targeted gas chromatography mass spectrometry (GC/MS) in conjunction with transcriptomics to assess the effect of ibuprofen on *Ae. aegypti* (Prud’homme et al., 2018). Direct ibuprofen exposure in larvae and adults had no effect on the 53 quantified polar metabolites, but F1 eggs from ibuprofen-exposed parents had lower levels of amino acids, carbohydrates, polyols, phosphoric acid, and ornithine, implying that ibuprofen exposure affected metabolic resource internalization in eggs (Prud’homme et al., 2018).

The profile of 12 amino acids and 31 acylcarnitines in *Culex quinquefasciatus* larvae treated with chlorpyrifos, temephos, and permethrin was determined using liquid chromatography tandem mass spectrometry (LC-MS/MS) (Martin-Park et al., 2017). Two acylcarnitines (C0 and C2) and the amino acid arginine were shown to be differentially associated with insecticide exposure. C0 concentrations were considerably higher in permethrin-exposed larvae, whereas C2 concentrations increased in permethrin-exposed but dropped in temephos-exposed larvae. Permethrin and temephos exposure enhanced arginine levels in larvae (Martin-Park et al., 2017). Studies have also shown that permethrin resistant mosquitoes have altered gut microbiota in comparison to the wild type and therefore have altered metabolic processes (Muturi et al., 2021).

In a separate study, *Culex pipiens* larvae subjected to varying concentrations of the neonicotinoid clothianidin showed differences in three groups of metabolites important in energy metabolism, including acylcarnitines, glycerophospholipids (GPs), and biogenic amine abundance (Russo et al., 2018) The unipolar and polar metabolites were quantified using flow injection tandem mass spectrometry (FIA-MS/MS) and LC-MS/MS, respectively. The highest dosage of clothianidin reduced acylcarnitines, GPs, and biogenic amines after 24 hours of exposure. Low and medium amounts reduced GPs and biogenic amines while increasing acylcarnitines. GPs and acylcarnitines were reduced at low and medium concentrations after 48 hours of exposure. These findings imply that low pesticide doses raised the energy requirements of exposed species (Russo et al., 2018).

Nuclear magnetic resonance (NMR) spectroscopy was utilized in *Ae. aegypti* to discover changes in metabolites caused by temperature and/or exposure to DDT, malathion, and deltamethrin. Metabolites involved in the tricarboxylic acid cycle, branched amino acid degradation, glycolysis/gluconeogenesis, amino acid, lipid and carbohydrate, nucleotide PRPP pathway, and phospholipid metabolism were the most affected (Singh et al., 2022). Seven of the nine discovered compounds were shown to be significantly altered by individual temperature and insecticide exposure. These included pyruvates, maltose, citrate, nicotinate, and -hydroxybutyrate, all of which are components of the glycolysis/gluconeogenesis, tri-carboxylic acid cycle energy producing pathways.

A recent study used LC-MS/MS to compare the metabolites of deltamethrin resistant and susceptible *An. sinensis* in larva and adult stages. Deltamethrin exposure resulted in the identification of 127 and 168 distinct metabolites in larvae and adults, respectively. Organooxygen compounds, carboxylic acids, GPs, and purine nucleic acids were shown to be different between resistant and susceptible mosquitos. The GPs route was shared by resistant larva and adults, and it may play an essential role in the metabolic deltamethrin detoxification (Li et al., 2022).

Pyrethroid resistance is typically associated with biological fitness costs, including size, longevity, fecundity, and mating behavior (Brito et al., 2013; Smith et al., 2016; Vera-Maloof et al., 2020). Moreover, pyrethroid resistance may influence the mosquito’s inherent permissiveness to viral infection, replication, and transmission. Few studies have reported the association between pyrethroid resistance and *Aedes* vectorial competence (VC), and the outcomes have been widely inconsistent among virus, mosquito strains and stage of viral infection. Few studies have evaluated the relationship between vector competence (VC) and pyrethroid resistance in laboratory *Ae. aegypti* (Zhao et al., 2018; Chen et al., 2020; Parker-Crockett et al., 2021; Stephenson et al., 2021; Wang L. et al., 2022) and *Ae. albopictus s*trains (Richards et al., 2017; Chen et al., 2021). The direction of VC response differed among virus, viral replication stage, and mosquito strains. For example, VC for ZIKV (Zhao et al., 2018; Parker-Crockett et al., 2021) and DENV (Chen et al., 2021) was higher in the pyrethroid resistant strain. In contrast, three investigations have shown that the pyrethroid-resistant strains had significantly lower viral infection rates than the susceptible strains (Deng et al., 2021; Stephenson et al., 2021; Wang L. et al., 2022). Interestingly, only one study found that high *kdr* allele frequencies were associated with lower DENV-infection rates in field populations in Florida (Stephenson et al., 2021). As more studies elucidate the specific interactions between arboviruses and field resistant mosquitoes, understanding the mechanisms of interaction in specific mosquito tissues remains to be explored.

### 6.2 The gut microbiome influences metabolic homeostasis in the mosquito

Recent studies on the mosquito microbiome have highlighted its critical impact on various stages of the mosquito life cycle including development and reproduction as well as ecological adaptation, pathogen infection, immunity and vector competence (**Figure 2A**). Increasing evidence suggests a critical metabolic inter-dependency between the microbiome and the mosquito vector that drive the resulting outcomes with detrimental effects if the metabolic relationship is perturbed.

The metabolic impact of the microbiome is evident throughout the life cycle of the mosquito. A comparison of *Ae. Aegypti* (requires a blood meal for egg production) versus *Ae. Atropalpus* (does not require a blood meal for egg production) identified that the microbiome played a crucial role in providing the nutritional resources required for *Ae. Atropalpus* egg production in the absence of a blood meal (Coon et al., 2016). Studies have also shown that bacterial microbiota in aquatic habitats significantly impact larval nutrient acquisition and/or assimilation influencing growth, development, and survival into pupation (Coon et al., 2017; Vogel et al., 2017; Correa et al., 2018; Valzania et al., 2018). These studies compared transcriptional responses in axenic (bacteria free) larvae and larvae containing either native or gnotobiotic (single species) microbiomes and discovered that processes such as amino acid transport, hormonal signaling, and metabolism were differentially expressed, suggesting a key role in larval development and survival. Axenic larvae exhibited delayed development time and stunted growth in comparison to their bacterially colonized counterparts.

A study by Feng et al., demonstrated the influence of gut microbiota of *Anopheles stephensi* (a potent malaria vector) on tryptophan metabolism. The study reported how elimination of gut microbiota *via* antibiotics increased accumulation of tryptophan and its metabolites (kynurenine and 3-hydroxykynurenine, 3H-K) in the mosquito with high levels of 3H-K leading to structural impairments in the peritrophic matrix which in turn facilitated *Plasmodium berghei* infection (Feng et al., 2021). Similar observations were also made by Das de et al (Das De et al., 2022),. Blood meal digestion also increased these specific metabolites in the midgut, and the gut microbiome was critical for the catabolism that maintains normal levels of these metabolites. In a second study, Bottino-Rojas et al., showed that the product of the kynurenine pathway, xanthurenic acid, was critical for controlling levels of microbiota as well as reproduction and survival of the mosquito (Bottino-Rojas et al., 2022). Mutations in orthologs of kynurenine hydroxylase impaired reproduction and survival in *Ae. aegypti*, *An. stephensi* and *Culex quinquefasciatus* as well as disrupted the midgut permeability barrier in *An. stephensi.* Kynurenine is a tryptophan metabolite. Together these studies suggest that tryptophan catabolism is critical for maintaining the integrity of the peritrophic matrix, preventing infection, maintaining normal levels of the gut microbiome, and ensuring survival and reproduction of the mosquito vector (Okech et al., 2006; Lima et al., 2012; Bottino-Rojas et al., 2022). Interestingly, tryptophan metabolism is a key pathway altered in the human metabolome during severe disease caused by DENV infection. Serotonin (another product of tryptophan metabolism) and kynurenine were identified to show differential abundance in DENV-infected patient serum. Serotonin levels were significantly lower in patients with dengue hemorrhagic fever (DHF) than those in the febrile phase (Cui et al., 2016).

On the other hand, there is evidence to show that metabolism of the mosquito influences the modulation of microbial density in different *Ae. aegypti* strains, in turn altering vector competence. A study by Short et al., demonstrated how specific metabolic pathways such as the branched chain amino acid (BCAA) degradation pathway can influence mosquito midgut microbiota. When the BCAA degradation pathway was silenced in two mosquito strains with broadly differing gut microbiota, a significant alteration in microbiota composition was observed in both strains. This resulted in levelling the variation between the two microbiomes. The authors hypothesize that variations in amino acid metabolism can be a crucial determinant of microbiota in a vector (Short et al., 2017). In relation to these results, there are previous studies that have shown how alterations of the mosquito midgut microbiota can alter vector competence in *Ae. aegypti* mosquitoes. An increase in DENV titers was observed in *Ae. aegypti* mosquitoes after reduction of bacterial loads in the midgut through antibiotic treatment (Xi et al., 2008). Moreover, introduction of multiple bacterial species into a native mosquito gut microbiome has been shown to decrease DENV titers (Ramirez et al., 2012; Ramirez et al., 2014). However, there is also evidence to show how specific midgut bacterial populations can enhance susceptibility of a vector to DENV and CHIKV (Apte-Deshpande et al., 2012; Apte-Deshpande et al., 2014). These studies highlight gaps that remain in our understanding of the mechanisms underlying the microbiome-mosquito-pathogen multipartite interactions that result in the outcomes observed, stimulating further studies.

### 6.3 Wolbachia as a control strategy impacts mosquito metabolism

As discussed earlier, vector control is an efficient way of preventing the spread of arboviruses. However, during the past two-three decades, the major vectors of these arboviruses have expanded their geographic range and population size (Kraemer et al., 2015). Rapid urbanization, climate changes and development of resistance to insecticides are some of the major factors governing the emergence of many medically important arboviruses. Efficient, vector control strategies are considered necessary to overcome these problems. In the last decade, interventions with biocontrol agents like *Wolbachia* have revolutionized the field of vector control. *Wolbachia* is a maternally inherited, endosymbiotic bacterium found naturally in ~ 60% of the insect population (Hilgenboecker et al., 2008; Zug and Hammerstein, 2012). Due to the unique capability of *Wolbachia* to block many arthropod-borne viruses, it has been widely used for biological control of mosquito borne viruses including dengue, West Nile and Zika (Moreira et al., 2009; Walker et al., 2011; Aliota et al., 2016). *Wolbachia* is a safe vector control strategy as there is no evidence of impact on the environment, animals or humans by the bacterium (Centers for Disease Control and Prevention, 2022). The mechanisms through which pathogen blocking is occurring are yet unknown. Understanding these mechanisms is critical to preclude any resistance development in the mosquitoes against the bacterium. Interestingly, there is evidence to show that *Wolbachia* alters metabolic homeostasis of the mosquito vector during infection (**Figure 2D**) (Caragata et al., 2014; Molloy et al., 2016; Koh et al., 2020; Manokaran et al., 2020; Nascimento da Silva et al., 2022). Given the reliance on metabolism for viral replication in the mosquito (discussed above), several studies have hypothesized that metabolic competition versus commensalism may be a mechanism for *Wolbachia*-mediated pathogen blocking.

Manokaran et al., showed that *Aag*2 cells infected with the *Wolbachia* (*w*Mel strain) had reduced abundances of acyl-carnitines which seemed detrimental for both DENV and ZIKV replication. Supplementation with acyl-carnitines restored DENV and ZIKV replication in *Wolbachia* infected cells (Manokaran et al., 2020). In the metabolomic study by Chotiwan, et al., acyl-carnitines were increased significantly in DENV2-infected mosquito midguts reinforcing the hypothesis that acyl-carnitines may play a proviral role in DENV replication and their regulation could be a possible mechanism of pathogen blocking by *Wolbachia.* Two reports exist for the intersection of SPs in insects and *Wolbachia*. Initially Rong et al. showed that *Wolbachia* infection induced the expression of miRNAs known to regulate genes with functions in sphingolipid metabolism (Rong et al., 2014). This was confirmed by Molloy et al, that recently showed a complete depletion of sphingolipids during *Wolbachia* infection of *Ae. albopictus* cells (Molloy et al., 2016).

Infection with *w*Mel has also been shown to influence oviposition, expression of egg yolk precursor genes as well as altered excretion of the blood meal in *Ae. aegypti* mosquitoes (Pimenta de Oliveira et al., 2017). The results of this study indicated delayed expression of genes required for development of eggs which in turn might have delayed yolk deposition creating a lag in oviposition. To further support their results, they call attention to studies by Caragata et al., that demonstrates how cholesterol levels were reduced by 15-25% in *Wolbachia* (wMelPop strain) infected *Ae. aegypti* mosquitoes (Caragata et al., 2014). Cholesterol is considered a primary precursor of the 20-hydroxyecdysone hormone that is an important regulator of oogenesis (Raikhel et al., 2002).

Competition for host resources such as cholesterol is also a proposed mechanism of pathogen blocking by *Wolbachia* (Caragata et al., 2013). The study conducted in *Wolbachia* (wMelPop and wMelICS) infected *Drosophila* flies fed with a cholesterol enriched diet has shown weakened pathogen blocking capabilities in comparison to flies fed with a standard diet. Both insect and virus are unable to synthesize their own cholesterol and depend on cholesterol taken up in the diet. Further, many of the arboviruses require host cholesterol to enter host cells and replicate (Mackenzie et al., 2007; Lee et al., 2008; Puerta-Guardo et al., 2010; Soto-Acosta et al., 2013; García Cordero et al., 2014). Thus, it is not surprising that manipulation of host cholesterol by *Wolbachia* could lead to perturbed viral entry and replication (Caragata et al., 2013). These observations are further supported by quantitative proteomics studies on *Aag*2 cells and *Ae. aegypti* midguts that revealed differential expression of proteins related to vesicular trafficking, lipid metabolism and unfolded protein response in response to *Wolbachia* infection (Geoghegan et al., 2017). Elevated levels of esterified cholesterol were detected in *Wolbachia* infected cells possibly resulting from perturbed intracellular cholesterol trafficking (Geoghegan et al., 2017). Reversal of this accumulation by treating with cyclodextrins restored dengue replication in these cells, suggesting free cholesterol was required for viral replication and sequestration of cholesterol through esterification seemed to enhance pathogen blocking. However, in this same study, Neiman Pick Type C1 and C2 (NPC1 and 2) proteins, responsible for retro recycling of cholesterol were elevated in expression compared to controls. Several studies have shown that elevated levels of NPC1 and 2 are beneficial to DENV replication. Therefore, the exact nature of the block in cholesterol trafficking and its relationship to NPC1 activity and viral replication remains to be explored further.

## 7 Conclusions

Living organisms display substantial diversity in morphology, anatomy, behavior, and ecology. In concert with this diversity, they are also analogous to each other based on fundamental molecular traits mirrored in metabolism and biochemical mechanisms of inheritance. Attributing to the fact that metabolites are universal molecules that are the intermediates and products of genetic expression, studying metabolism to answer fundamental biological questions related to changes in physiological status in an organism in response to external stimuli or infection is becoming a promising avenue to explore. Among numerous pathogens imposing health burdens on global populations, arboviruses occupy a prominent position due to their causation of severe disease with a lack of successful intervention approaches. Many arboviruses such as dengue, Zika and chikungunya are vectored primarily by mosquitoes. Even though mosquito biology has been studied for many years, severe gaps remain in our understanding of the complex virus-vector interactions that continue to support disease transmission. Given the intimate connections between vector metabolism and its biology as well as the metabolic impact of pathogens and the microbiome on mosquito biology, the literature reviewed here suggest that exploiting metabolism-targeted intervention strategies will be transformative.

## Author contributions

OR, NC, KS and RP wrote the manuscript. OR and RP assembled the figures and tables. All authors contributed to the article and approved the submitted version.
